# Advanced hyperspectral image processing and machine learning approaches for early detection of wheat stem rust

**DOI:** 10.3389/fpls.2025.1725017

**Published:** 2025-12-18

**Authors:** Alexander Fedotov, Danila Eremenko, Daria Kuznetsova, Olga Baranova, Anton Terentev

**Affiliations:** 1 Peter the Great St. Petersburg Polytechnic University, Saint Petersburg, Russia; 2 All-Russian Institute of Plant Protection, Saint Petersburg, Russia

**Keywords:** early plant disease detection, explainable ML, feature importance, hyperspectral data processing, machine learning, preprocessingpipeline, wheat (*Triticum aestivum* L.), wheat stem rust (*Pucciniagraminis* f. sp. *tritici*)

## Abstract

Hyperspectral remote sensing has shown great promise for early detection of plant diseases, yet its adoption is often hindered by spectral variability, noise, and distribution shifts across acquisition conditions. In this study, we present a systematic preprocessing pipeline tailored for hyperspectral data in plant disease detection, combining pixel-wise correction, curve-wise normalization and smoothing, and channel-wise standardization. The pipeline was evaluated on an experiment on early detection of stem rust (*Puccinia graminis* f. sp. *tritici* Eriks. and E. Henn.) of wheat (*Triticum aestivum* L.). The pipeline implementation enhanced the classification models accuracy raising F1-scores of logistic regression, support vector machines and Light Gradient Boosting Machine from 0.67–0.75 (raw spectra) to 0.86–0.94. Notably, it enabled reliable detection of asymptomatic infections as early as 4 days after inoculation, which was not achievable without preprocessing. The framework demonstrates potential for generalization beyond plant pathology, suggesting applicability to a range of hyperspectral remote sensing tasks such as vegetative health monitoring, environmental assessment, and material classification through improved signal interpretability and robustness. This work lays the groundwork for advancing hyperspectral image processing by proposing a reproducible, scalable pipeline that could be adapted for integration into unmanned and satellite imaging systems.

## 1 Introduction

Hyperspectral remote sensing is a powerful technique for extracting detailed information from various objects by measuring reflected electromagnetic radiation across multiple spectral bands in the UV (Ultraviolet), VIS (Visible Spectrum), NIR (Near-Infrared), and SWIR (Short-Wave Infrared) ranges (Pu, 2017). It is widely applied in fields such as astronomy, geology, medicine, quality control, and environmental monitoring (Khan et al., 2022; Stuart et al., 2019; Meher and Panda, 2021). In recent years, it has gained attention for its applications in disease diagnostics and material analysis (Gao and Smith, 2015; Grzybowski et al., 2021; Yoon, 2022). One emerging direction is early plant disease detection, which represents a challenging classification problem due to complex spectral variability (Zhang et al., 2020; Shahi et al., 2023).

Wheat is a major global crop with annual production exceeding 770 million tons (Fróna et al., 2019; FAO, 2025);. Various biotic and abiotic factors may contribute to significant wheat yield decrease. The stem rust, caused by the biotrophic fungus *Puccinia graminis* f. sp. *tritici* Eriks. and E. Henn. (*Pgt*), is one of the most harmful wheat diseases. The disease mainly affects stems and leaf sheaths, sometimes harming the leaves, awns and glumes of the plant. A brown-rusty or reddish-brown pustules containing a powdery mass of urediniospores are formed on the infected plants. The first visible pustules appear on the 8th day after infection. They release the urediniospores between the 10th and 15th day. The disease increases transpiration and disturbs the water balance. Severe infection of stems interrupts the flow of nutrients to the developing ears, which leads to the grain shriveling. A large number of pustules which merge with each other leads to ruptures of the epidermis and then to the host plant lodging (Leonard and Szabo, 2005). Yield losses for susceptible varieties can reach up to 100% (Ashagre, 2022). Healthy wheat that is three weeks away from harvest can be lost if sufficient inoculum is carried by air masses from distant regions (Leonard and Szabo, 2005). The emergence of virulent aggressive races of stem rust, such as the famous Ug99 race (TTKSK), which appeared in 1999 in Uganda (Pretorius et al., 2000) and affected wheat varieties with the *Sr31* gene, which provided protection against all races of the fungus before the emergence of Ug99, has increased the concern of grain producers worldwide. To date, the Ug99 race of stem rust and its biotypes have spread across the African continent and the Middle East. On the other hand, non-Ug99 races of the fungus, such as TKKTP, TKTTF, TTTTF, have spread across the Eurasian continent (Lewis et al., 2018; Patpour et al., 2022; Baranova et al., 2023). They are avirulent to *Sr31*-protected varieties but cause severe damage to susceptible varieties. It has been shown that 55% of North American and international varieties and breeding lines resistant to the TTKSK race (Ug99) are susceptible to the TKKTP race (Olivera Firpo et al., 2017). The TKTTF race has been found in the Middle East and Europe, including the UK. The TTTTF race of stem rust affected several thousand hectares of durum wheat in Sicily in 2016 (Lewis et al., 2018). The fungus *P. graminis* f. sp. *tritici* is a basidiomycete fungus with a complex development cycle - asexual reproduction occurs on the main host plant - wheat, and the sexual stage - on the alternate host - *Berberis* spp. The mycelium of the fungus spreads in plant tissues along intercellular spaces, and haustoria penetrate cells from which the fungus receives nutrients. The huge number of urediniospores produced by the fungus, their ability to be carried with air masses over long distances and susceptibility to the stem rust of most commercial varieties of wheat makes this pathogen a serious threat to food security. The fungus is successfully destroyed by fungicides, but chemical control measures are only effective when applied early, thus rapid and non-invasive diagnostic methods are essential. Hyperspectral remote sensing offers significant potential in this regard.

The current state of the art in hyperspectral diagnostics and plant disease monitoring is well developed. Recent comprehensive reviews highlight a wide range of studies addressing these topics, in which various mathematical and computational tools have been applied (García-Vera et al., 2024). These approaches include vegetation indices (Radočaj et al., 2023), statistical algorithms (Wan et al., 2022; García-Vera et al., 2024), machine-learning techniques (Grewal et al., 2023; García-Vera et al., 2024), and deep-learning methods (Grewal et al., 2023; Guerri et al., 2024; Thapa et al., 2024).

Despite this considerable progress, the number of studies focused specifically on early hyperspectral disease detection remains limited (Terentev et al., 2022). Most existing works rely on simplified or fully controlled experimental settings, and the applied algorithms are often optimized for particular datasets, which constrains their transferability and reproducibility (Li et al., 2021; Wan et al., 2022). Such experimental conditions frequently overlook key remote-sensing challenges encountered in real agricultural environments, including uneven illumination, overlapping canopy structures, background noise, and day-to-day variability in acquisition conditions (Qi et al., 2023; Zhang Z. et al., 2024).

However, despite rapid methodological advances, preprocessing variability in hyperspectral plant disease detection remains insufficiently explored. A major gap in current research is the absence of systematic comparisons among different preprocessing strategies (pixel-wise, curve-wise, and channel-wise) and their combined impact on model generalization across varying class distributions. Many previous studies have implemented single-step normalization or heuristic feature extraction without quantitatively assessing how these steps influence classification stability, particularly in asymptomatic infection stages. Consequently, preprocessing workflows are often under-described or inadequately detailed for reliable replication. Nevertheless, recent studies emphasize that a well-designed preprocessing pipeline is essential for robust hyperspectral analysis (Li et al., 2021; Cozzolino et al., 2023).

Research on wheat stem rust (*P. graminis* f. sp. *tritici*) remains scarce, with only a few studies addressing this pathogen directly (Abdulridha et al., 2023; Terentev et al., 2025). While several works have explored leaf rust (*P. triticina* Erikss.) (Bohnenkamp et al., 2019; Terentev et al., 2023), most focus on yellow rust (*P. striiformis* Westend), currently the most prevalent and damaging member of the *Puccinia* genus (Guo et al., 2020, 2021; Huang et al., 2022; Deng et al., 2023; Ren et al., 2025). Nevertheless, stem rust also poses a major threat to wheat production (Ashagre, 2022; Willocquet et al., 2022). Given the substantial physiological and biochemical differences among *Puccinia* species - and consequently in their spectral responses - stem rust was selected in this study as a model disease that remains under-investigated in hyperspectral imaging research (Singh et al., 2002; Kolmer, 2005).

In our previous work, we demonstrated the feasibility of early wheat leaf rust detection using hyperspectral imaging of detached leaves under controlled conditions (Terentev et al., 2023). In the present study, we extend this approach to intact potted plants under field-simulated conditions - a standard experimental setup for investigating biotrophic pathogens (Kolmer et al., 2007; Kolmer, 2019).

Here, we systematically evaluate how different hyperspectral preprocessing strategies -including pixel-wise, curve-wise, and channel-wise normalization and smoothing - affect the accuracy, robustness, and feature-space separability of machine-learning models for detecting wheat plants infected with *P. graminis* f. sp. *tritici* at early, asymptomatic stages under field-simulated conditions. We present and validate a modular preprocessing framework for hyperspectral early-disease detection. This framework provides early, accurate, and interpretable detection performance under real-world variability and class imbalance. The classification task is performed at the plant level, distinguishing healthy versus diseased samples.

The objective of this study was to develop, test, and validate a modular hyperspectral preprocessing pipeline that ensures methodological transparency and reproducibility, while providing practical guidance for building robust machine-learning systems capable of operating effectively in dynamic agricultural environments. The novelty of this study lies in the first systematic comparison of pixel-, curve-, and channel-wise preprocessing strategies for early plant disease detection, demonstrated here using *Pgt* -inoculated wheat plants.

## 2 Materials and methods

### 2.1 Data preprocessing and preparation

The study used soft spring wheat (*Triticum aestivum* L.) of the “Saratovskaya 74” variety, which is susceptible to *P. graminis* f. sp. *tritici*. Wheat was sown in plastic containers measuring 320x220x160 mm, 75 seeds per container. Optimal care conditions (photoperiod, watering, fertilization) were maintained for the plants. A suspension of *Pgt* urediniospores from the collection of the Laboratory of Plant Immunity to Diseases, All Russian Institute of Plant Protection, was used for inoculation at a concentration of 1 mg spores/ml. Eleven-day-old wheat seedlings (stage 12 on the BBCH scale) were inoculated with a spore suspension under laboratory conditions with NOVEC 7100 hydrofluoroether additive, using an airbrush. (Baranova et al., 2023; Terentev et al., 2025) and then placed in a dark chamber with 100% humidity at a temperature of 23°C for 24 hours. Afterward, the plants were transferred to a phytotron at 25°C with an 18-hour photoperiod at 250–270 PPFD (Photosynthetic Photon Flux Density) illumination and 60% humidity for the following 10 days. Visible signs of stem rust infection appeared between the 6th and 10th day following inoculation. Disease evaluation was conducted according to standard laboratory protocols for seedling testing (Jin et al., 2007). By the 10th day, the infection type reached level 4 on the conventional Stakman 0–4 scale (Stakman et al., 1962). The intensity of disease development was quantified using the modified Cobb scale after Peterson (Peterson et al., 1948), giving severity values between 70 and 90 across both experimental repetitions.

Hyperspectral imaging was carried out in a light-insulated chamber. The camera was positioned horizontally on a tripod at a height of 0.5 m above the plant samples. Illumination was provided by two 500 W halogen lamps placed at 45° angles. A dark, non-reflective background was used to optimize the contrast of plant tissue (Zhang X. et al., 2024). The imaging field covered an area of 20 × 20 cm. The experimental layout followed previously validated imaging setups (Zhu et al., 2016; Wang et al., 2018; Gu et al., 2019). The plants were imaged as if they were in the field, i.e., from above.

The experiments utilized an Ultris 20 hyperspectral snapshot camera (Cubert GmbH, Ulm, Germany) operating in the 450–874 nm spectral range, comprising 106 bands with 4 nm intervals. Images were captured at a spatial resolution of 410 × 410 pixels. Calibration included dark and white calibration and geometric distance adjustment using the calibration standards provided by camera manufacturer and Cubert Pilot software (version 2.8.1, Cubert GmbH, Ulm, Germany). Two independent datasets were collected. Each dataset consisted of 864 hyperspectral cubes, acquired in period between days 4 and 9 after pathogen inoculation. Each daily dataset (the control group, which consisted of healthy plants, and the experimental group which consisted of stem rust inoculated plants) contained 144 images. Each complete dataset comprised 864 images (432 healthy, 432 diseased). Data were saved in Multi-Channel TIFF format (106 channels, 16-bit). Each hyperspectral snapshot was represented as 3-dimensional matrix $H^{s}$ of shape 410 x 410 x 106 where 410 are height and width of hyperspectral image and 106 is a number of hyperspectral channels.

All computations were performed in Python 3.10 using scikit-learn, Optuna, SciPy, Pandas, and Matplotlib/Seaborn on a workstation equipped with an Intel Core i5-12400F CPU, 32 GB RAM, and an NVIDIA GeForce 3070 Ti GPU.

#### 2.1.1 Research layout

**Figure 1:**
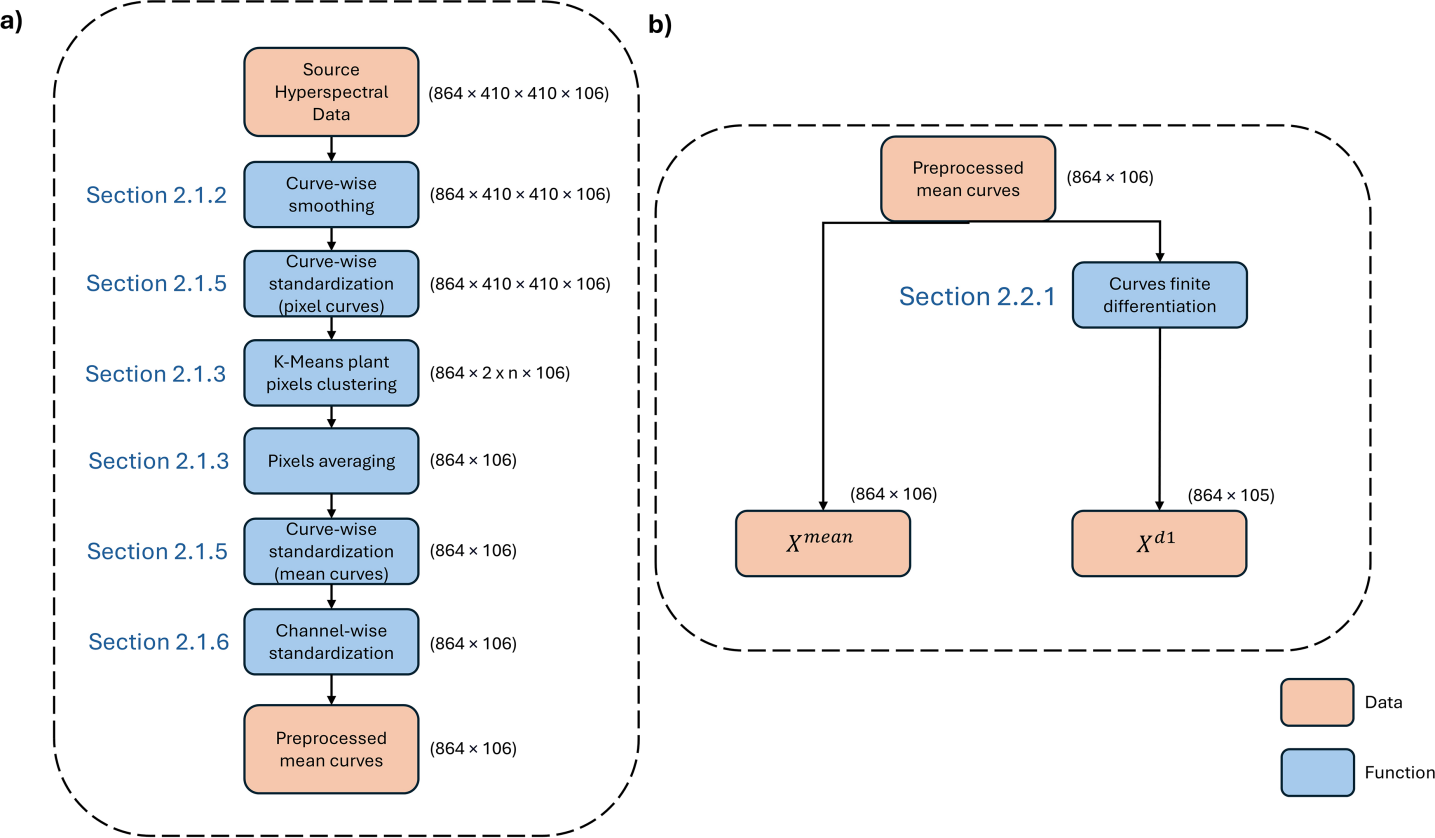
Research layout. **(a)** sequential preprocessing steps, **(b)** the algorithms used for feature extraction from hyperspectral curves.

**Figure 1** illustrates the research layout. **Figure 1a** illustrates the sequential application of preprocessing steps and the dimensionality reduction of the original data after each preprocessing stage. And **Figure 1b** illustrates the algorithms used for feature extraction from hyperspectral curves.

#### 2.1.2 Curve-wise smoothing

To smooth hyperspectral curves and filter possible noise we used Savitzky-Golay filter (Savitzky and Golay, 1964; Rinnan et al., 2009) which is a method of constructing a sliding window data approximation using a polynomial of *k-th* degree. Formally the method can be described as follows:


$$p_{N}(x)=\sum_{n=0}^{p^{savgol}}c_{n}x^{n}=c_{0}+c_{1}x+\ldots +c_{n-1}x^{n-1}+c_{n}x^{n}$$

Where x – is a point of hyperspectral curve and $c_{n}$ is coefficients of polynom of $p^{savgol}$ degree.

Optimization function for each sliding window is a sum of squared errors minimization between the datasets consisting of *W* points between the hyperspectral curve and values of approximation polynomial $p_{N}$ inside each window:


$$\sum_{i=-w^{savgol}}^{2w^{savgol}}\;{\left[p_{N}(x_{i})-x_{i}\right]}^{2}\to min$$

Varied preprocessing hyperparameters are $p^{savgol}$ (polynomial order for each window approximation) and $w^{savgol}$ (length of sliding window). A more detailed description of hyperparameters is provided in **Table 1** in Section 2.3.3. The following smoothing algorithm implementation from the SciPy library was used in the study (Virtanen et al., 2020).

**Table 1:** Preprocessing scenarios for each model.

Preprocessing	Parameter	Values
pixel-wise preprocessing (pixel level)	method	*none, minmax, standard, robust*
pixel-wise smoothing	$p^{savgol}$	*1, 2, 3, 4, 5*
	$w^{savgol}$	*3, 5, 7, 9, 11, 13, 15*
curve-wise preprocessing (mean curves level)	method	*none, minmax, standard, robust*
channel-wise deviation preprocessing	method	*none, minmax, standard, robust*
	grouping strategy	*all, datasets, days*

Italic values denote mathematical notation for parameters, variables, and distribution functions.

#### 2.1.3 K-Means clustering

**Figure 2:**
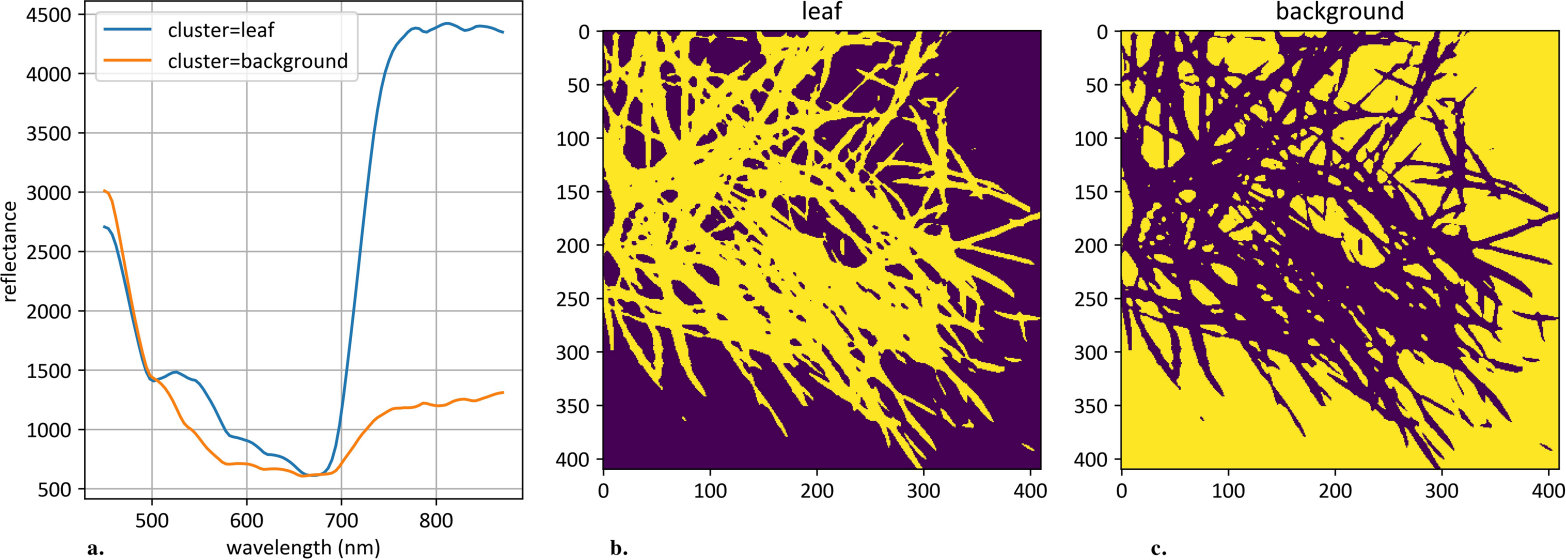
An example of hyperspectral image clustering. **(a)** demonstrates mean curves generated from plant and background pixels. The vertical scale shows the reflectivity expressed in absolute values, and the horizontal scale shows the spectrum wavelengths expressed in nanometers. **(b)** demonstrates an example of clustered plant pixels, highlighted in yellow. **(c)** demonstrates an example of clustered background pixels, highlighted in yellow.

Clustering was applied to the 106-dimensional hyperspectral pixel space to segment objects on each hyperspectral image. The K-Means algorithm was used for this task with a predefined number of clusters, set to 2 (Hartigan and Wong, 1979). Cluster identification was performed based on the following set of rules: (1) the cluster with the minimum integral reflectance was classified as background; (2) the cluster with the maximum reflectance deviation was classified as plant object. After clustering each individual image, an average curve according to plant pixels was calculated. An example of such clustering is shown in **Figure 2**.

Formally, object segmentation using clustering and mean curve calculation across all hyperspectral pixels belonging to an object can be described as follows:

Let us introduce hyperspectral pixels $P^{s}\;$corresponded to the plant object on the *s-th* hyperspectral snapshot as follows:


$$P^{s}=\{p\in H^{s}\;|\;k_{s}(p)=\zeta^{smax}\}$$

Here $k_{s}$ is prediction function of K-Means algorithm fitted on the $H^{s}$, $\zeta^{smax}$ is an index of cluster with maximum deviation on the $H^{s}$ and p is hyperspectral pixel represented as a 106-dimensional vector.

Let us introduce $X^{mean}$ as a matrix that consists of 864 vectors of length 106 representing mean hyperspectral curves received via averaging all individual hyperspectral pixel corresponded to the object:


$$x_{s,c}^{mean}=\;\frac{\sum_{p\in P^{s}}p_{c}}{\left|P^{s}\right|}$$

Here $x_{s,c}^{mean}$ is an element of matrix $X^{mean}$ representing mean value of plant pixel on the *s-th* snapshot in *c-th* hyperspectral channel.

#### 2.1.4 Source hyperspectral curves

**Figure 3:**
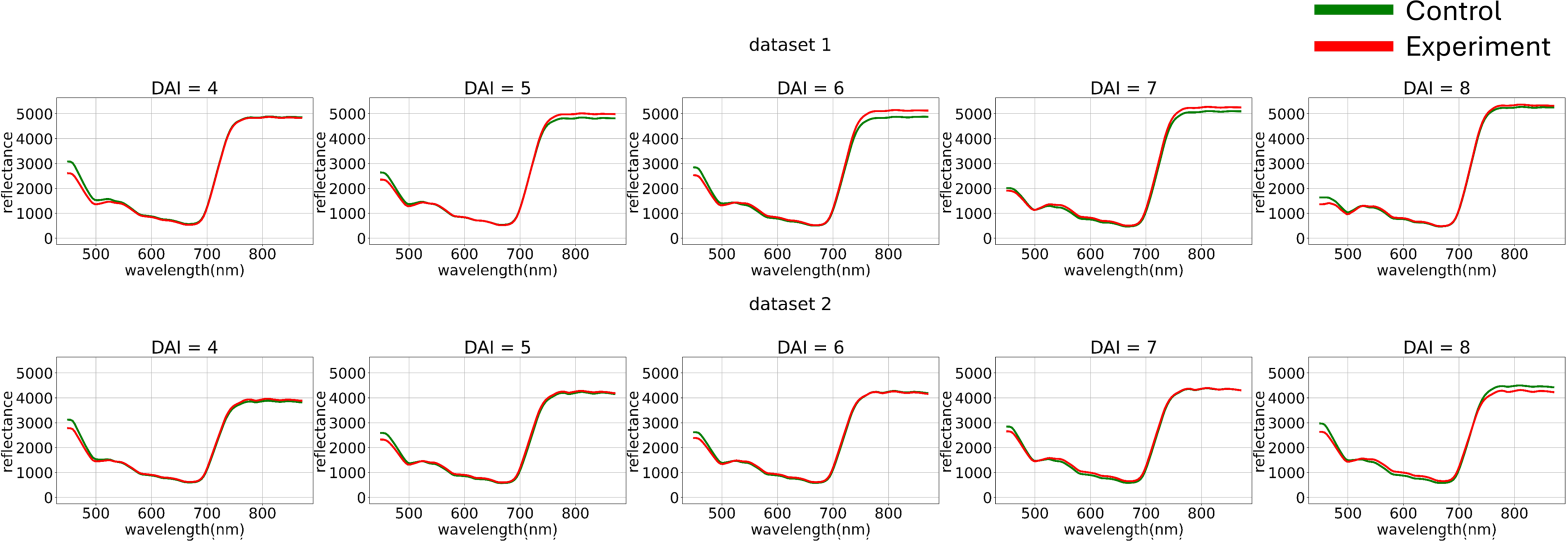
Average hyperspectral curves of the control and experimental groups from 4 (DAI = 4) to 8 (DAI = 8) Days After Inoculation across both imaging sessions (raw data) (top – first repetition of the experiment, bottom – second repetition of the experiment). Control - healthy wheat plants, Experiment - wheat plants inoculated with *Pgt*. The vertical scale shows the reflectivity expressed in absolute values, and the horizontal scale shows the spectrum wavelengths expressed in nanometers.

After image clustering, a comparative analysis of average curves across daily subsets of the original dataset was conducted (**Figure 3**).

**Figure 3** illustrates how preliminary visualization demonstrated a similar distinction between classes in the blue range, specifically that the curves of diseased plants exhibit lower reflectance.

#### 2.1.5 Curve-wise standardization

**Figure 4:**
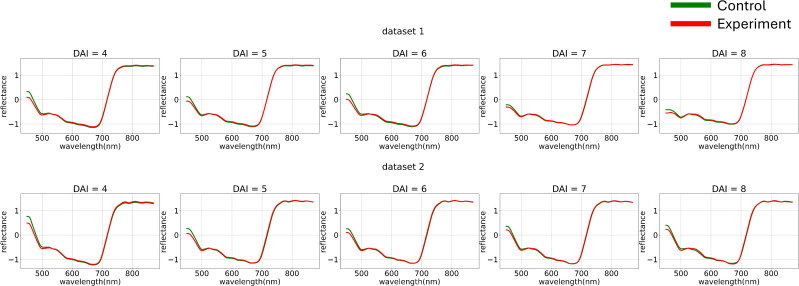
Average hyperspectral curves of the control and experimental groups from 4 (DAI = 4) to 8 (DAI = 8) days after inoculation across both imaging sessions (after applying curve-wise standard preprocessing) (top – first repetition of the experiment, bottom – second repetition of the experiment). Control - healthy wheat plants, Experiment - wheat plants inoculated with *Pgt*. The vertical scale shows the reflectivity expressed in normalized values, and the horizontal scale shows the spectrum wavelengths expressed in nanometers.


To minimize noise impact on the data, various normalization functions were applied to each individual curve (**Figure 4**). The normalization of each element of matrix $X^{mean}$ can be described as follows:

$$f^{cuminmax}(x_{s,c}^{mean})=\;\frac{x_{s,c}^{mean}-\text{min}(x_{s}^{mean})}{\max(x_{s}^{mean})-\text{min}(x_{s}^{mean})}$$

$$f^{custandard}(x_{s,c}^{mean})=\;\frac{x_{s,c}^{mean}-\text{mean}(x_{s}^{mean})}{std(x_{s}^{mean})}$$

$$f^{curobust}(x_{s,c}^{mean})=\;\frac{x_{s,c}^{mean}-\text{median}(x_{s}^{mean})}{\text{q}3(x_{s}^{mean})-\text{q}1(x_{s}^{mean})}$$

Where $f^{cuminmax}$- curve-wise minmax preprocessing function, $f^{custandard}$ - curve-wise standard preprocessing function, $f^{curobust}$ - curve-wise robust preprocessing function.

#### 2.1.6 Channel-wise standardization

Channel-wise deviation equalization using different statistical methods can formally be described as follows:


$$f^{chminmax}(x_{s,c}^{mean})=\frac{\left(x_{s,c}^{mean}-\;min^{c}\right)}{max^{c}-min^{c}}\cdot \text{max}(\{max^{c}-min^{c}\;|\;c\in 1..106\})$$

$$f^{chstandard}(x_{s,c}^{mean})=\frac{\left(x_{s,c}^{mean}-\;\mu^{c}\right)}{\sigma^{c}}\cdot \text{max}(\{\sigma^{c}|\;c\in 1..106\})$$

$$f^{chrobust}(x_{s,c}^{mean})=\frac{\left(x_{s,c}^{mean}-\;median^{c}\right)}{q3^{c}-q3^{c}}\cdot \text{max}(\{q3^{c}-q3^{c}\;|\;c\in 1..106\})$$

Where $\mu^{c}$, $median^{c}\;$, $\sigma^{c}$, $min^{c}$, $\;max^{c}$ are mean, median, standard deviation, minimum and maximum of sum subset of collected dataset depends on standardization strategy by *c-th* hyperspectral channel. And $f^{chminmax}$ is channel-wise minmax preprocessing function, $f^{chstandard}$ - channel-wise standard preprocessing function, $f^{chrobust}$ -channel-wise robust preprocessing function.

**Figure 5:**
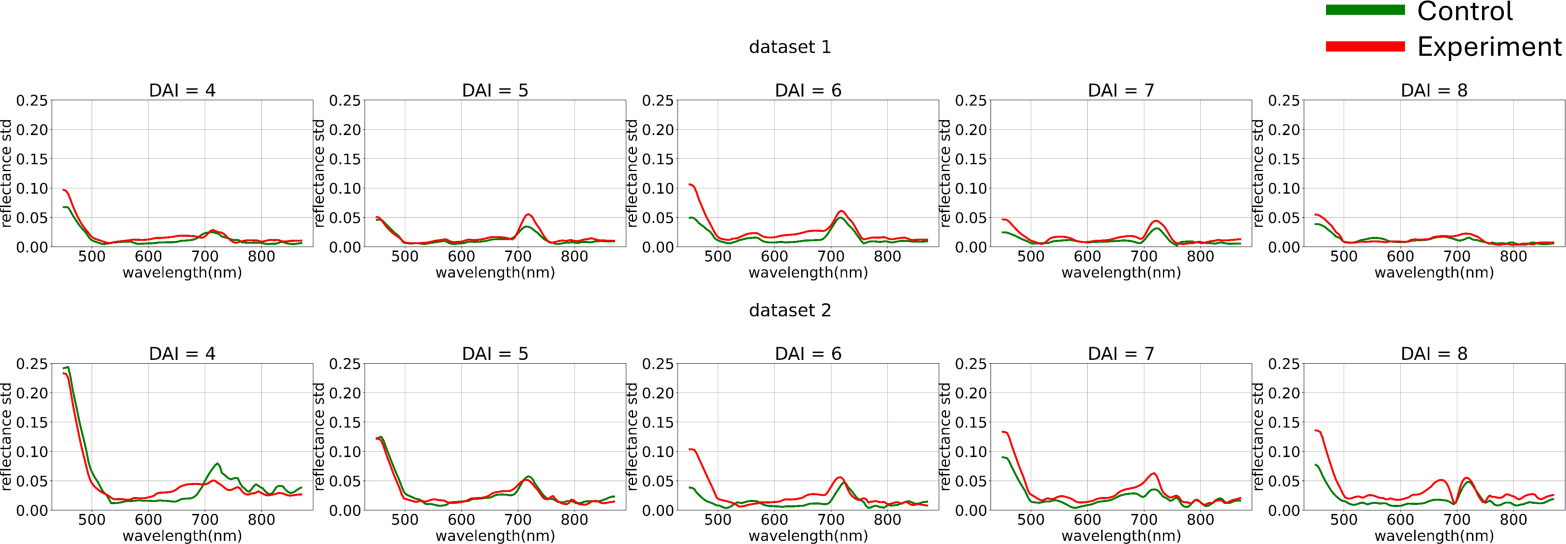
Average hyperspectral curves of the control and experimental groups from 4 (DAI = 4) to 8 (DAI = 8) days after inoculation across both imaging sessions (after applying curves channel-wise variance preprocessing) (top – first repetition of the experiment, bottom – second repetition of the experiment). Control - healthy wheat plants, Experiment - wheat plants inoculated with *Pgt*. The vertical scale shows the reflectivity expressed in normalized values, and the horizontal scale shows the spectrum wavelengths expressed in nanometers.

**Figure 5** illustrates the variance differences between channels in both experiments. The blue range showed higher variance compared to the green and red ranges. To better analyze curve shapes, the variance of each channel was standardized to a uniform value across the entire dataset.

At this stage, we used non-robust standardization to visually demonstrate the channel-wise variance impact. However, the choice of variance equalization method is also treated as a model training hyperparameter.

The next task was to select a methodology for splitting the original dataset into subsets for standardization. In this study, we applied three strategies for channel-wise variance equalization: using the entire dataset, treating each experiment separately, and treating each day within each experiment separately. Therefore, the strategy for channel-wise variance equalization became another model training hyperparameter. Let’s denote this hyperparameter as the “*grouping strategy*” with the following values: *all* – normalization of the entire dataset without grouping, *dsets* – normalization with grouping by experiments, and *days* – normalization with grouping by days and experiments.

**Figure 6:**
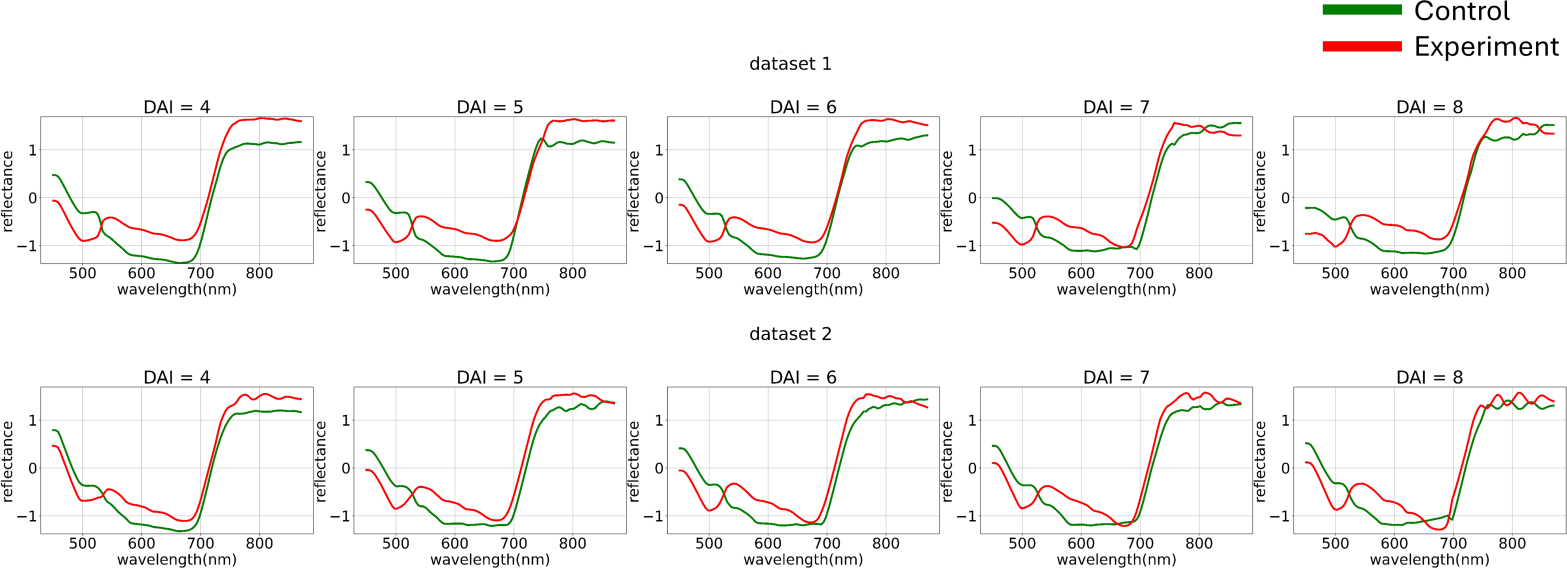
Average hyperspectral curves of the control and experimental groups from 4 (DAI = 4) to 8 (DAI = 8) days after inoculation across both imaging sessions (after applying channel-wise standard normalization preprocessing) (top – first repetition of the experiment, bottom – second repetition of the experiment). Control - healthy wheat plants, Experiment - wheat plants inoculated with *Pgt*. The vertical scale shows the reflectivity expressed in normalized values, and the horizontal scale shows the spectrum wavelengths expressed in nanometers.

**Figure 6** shows the hyperspectral curves after applying channel-wise standardization and variance equalization. After standardizing the variance of each channel and visualizing the average curves, the following trend was observed: when examining the control group’s curves as a dynamically changing function, it becomes apparent that from 440 nm to 700 nm, the curve behaves almost monotonically. The curve for the diseased plant, on the other hand, displays a section with a positive derivative from 500 nm to 530 nm within the same range. This observation suggests the applicability of methods analyzing the dynamics of hyperspectral curves.

### 2.2 Feature extraction

#### 2.2.1 Curves finite differentiation

**Figure 7:**
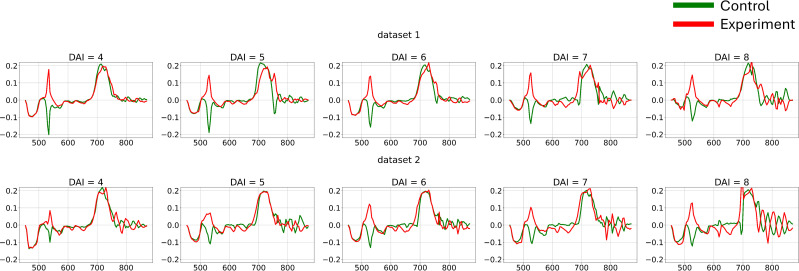
Average hyperspectral curves of the control and experimental groups from 4 (DAI = 4) to 8 (DAI = 8) days after inoculation across both imaging sessions (first-derivatives) (top – first repetition of the experiment, bottom – second repetition of the experiment). Control - healthy wheat plants, Experiment - wheat plants inoculated with *Pgt*. The vertical scale shows the reflectivity expressed in normalized values, and the horizontal scale shows the spectrum wavelengths expressed in nanometers.

The differences in channel-wise dynamics of hyperspectral curve changes identified in the previous section provide a basis for applying various methods to extract shape information from hyperspectral data for additional feature generation. To capture the dynamic changes in hyperspectral curves and create a new feature space for each individual curve, first-order derivatives were calculated (**Figure 7**).

Let us define $X^{d1}$ as a matrix consists of 864 vectors of length 105 representing first derivatives of mean hyperspectral curves $X^{mean}$


$$x_{s,c}^{d1}=x_{s,c+1}^{mean}-x_{s,c}^{mean}$$

### 2.3 Machine learning pipeline

#### 2.3.1 Machine learning models

Three machine learning algorithms were used to analyze the preprocessed data: Support Vector Machine (SVM) (Cortes and Vapnik, 1995), Logistic Regression (LR) (Hosmer and Lemeshow, 2000), and Light Gradient Boosting Machine (LGBM) (Ke et al., 2017) which is an extension of gradient boosting algorithm (Friedman, 2002). SVM was applied for hyperspectral data classification due to its ability to construct an optimal separating hyperplane and use kernel functions for linearly non-separable data. LR served as a baseline linear model for comparison. LGBM implements gradient boosting with Gradient-based One-Side Sampling (GOSS) and Exclusive Feature Bundling (EFB) optimizations to improve training speed and handle a large number of features.

Hyperparameters tuning space (regularization, kernels, tree depth, learning rate, etc.) is described in Section 2.3.3. The model selection was based on their proven efficiency in hyperspectral data classification in previous studies.

#### 2.3.2 Train and validation methodology

The cross-testing scenarios that were used in this study are shown in the **Table 2** below. In this approach, training and validation were performed on the data from one experiment, while testing was conducted on the data from the other experiment each time.

**Table 2:** Cross-testing scenarios.

Train dataset	Test subset
Dataset 1	Dataset 2
Dataset 2	Dataset 1

Model hyperparameters were selected using a cross-validation algorithm (**Table 3**). The folds for cross-validation were based on the experiment days, as shown in the table below. The cross-validation algorithm criterion was the highest mean AUC (Area Under the Curve) across all folds.

**Table 3:** Cross-validation scenarios.

Train subset (DAI)	Validation subset (DAI)
4, 5	6, 7
6, 7	4, 5
4, 5	7, 8
7, 8	4, 5

#### 2.3.3 Models hyperparameters

We performed hyperparameter tuning using the Tree-structured Parzen Estimator algorithm implemented in the Optuna library (Akiba et al., 2019), running the optimization for 200 iterations (**Table 4**). In the **Table 4***loguniform(low, high)* is a function that generates values in accordance with the reciprocal or log-uniform over the [low, high] interval and *randint(low, high)* is a function that generates integer values uniformly over the *[low, high]* interval.

**Table 4:** The hyperparameter tuning space.

Model	Implementation/ parameters description	Parameter	Search space
LR	https://scikit-learn.org/stable/modules/generated/sklearn.linear_model.LogisticRegression.html	penalty (strategy of regularization)	*l1, l2*
		C (regularization strength)	*loguniform(1e0, 1e6)*
		solver (optimization algorithm)	LIBLINEAR (Fan et al., 2008), SAGA (Defazio et al., 2014)
SVM	https://scikit-learn.org/stable/modules/generated/sklearn.svm.SVC.html	kernel (strategy of additional internal feature generation)	*Linear, rbf, poly*
		C (regularization strength)	*loguniform(1e0, 1e6)*
LGBM	https://lightgbm.readthedocs.io/en/stable/Parameters-Tuning.html	n_estimators (number of decision trees)	*randint(1, 700)*
		learning rate (weight of each tree in sequence)	*loguniform(1e-5, 1e0)*
		lambda_l1 (L1 regularization)	*loguniform(1e-3, 1e3)*
		lambda_l2 (L2 regularization)	*loguniform(1e-3, 1e3)*
		max_depth (maximum tree depth)	*randint(2, 10)*

Italic values denote mathematical notation for parameters, variables, and distribution functions.

**Table 4** presents the hyperparameters of each model and their distributions used for each search strategy.

Additionally, for each model, a full enumeration of the various preprocessing methods described in sections 2.1.3 to 2.1.5 was performed (**Table 1**).

#### 2.3.4 Test and validation metrics

The testing and validation of the models were based on the metrics listed in **Table 5**.

**Table 5:** Test and validation metrics.

Performance indicators	Designations
$Recall=\frac{TP}{TP+FN}$ – the proportion of correctly predicted positive classes among all true positive classes.	TP – number of true-positive predictions.
	FN – number of false-negative predictions.
$Precision=\frac{TP}{TP+FP}$ – the proportion of correctly predicted positive classes among all predicted positive classes.	TP – number of true-positive predictions.
	FP – number of false-positive predictions.
$F1=2\times \frac{Precision\times Recall}{\left(Precision+Recall\right)}$ mean of Precision and Recall	

## 3 Results

This section presents the experimental results obtained from applying the proposed preprocessing strategies and classification approaches to hyperspectral images of wheat plants under field-simulated conditions. The primary objective was to evaluate how different normalization methods, smoothing techniques, and feature representations influence the performance of machine learning models in early disease detection. To achieve this, we first analyzed the effect of preprocessing on data consistency and spectral quality, then assessed the impact of these transformations on classification accuracy across different feature spaces. Subsequent subsections compare various machine learning algorithms, explored the role of preprocessing at curve-wise and channel-wise levels, and examined model robustness under distribution shifts and varying illumination conditions. Finally, we investigated the contribution of specific spectral ranges to classification outcomes.

### 3.1 The biological experiment results

In nature, the stem rust pathogen development is favored by hot days and warm nights with favorable temperatures of 15-20°C at night and 25-30°C during the day, as well as sufficient humidity, which is facilitated by rain or dew leaving drops on plant leaves (Schumann and Leonard, 2000). In our experiment, controlled conditions were maintained for the inoculation and growth of *P. graminis* f. sp. *tritici*.

**Figure 8:**
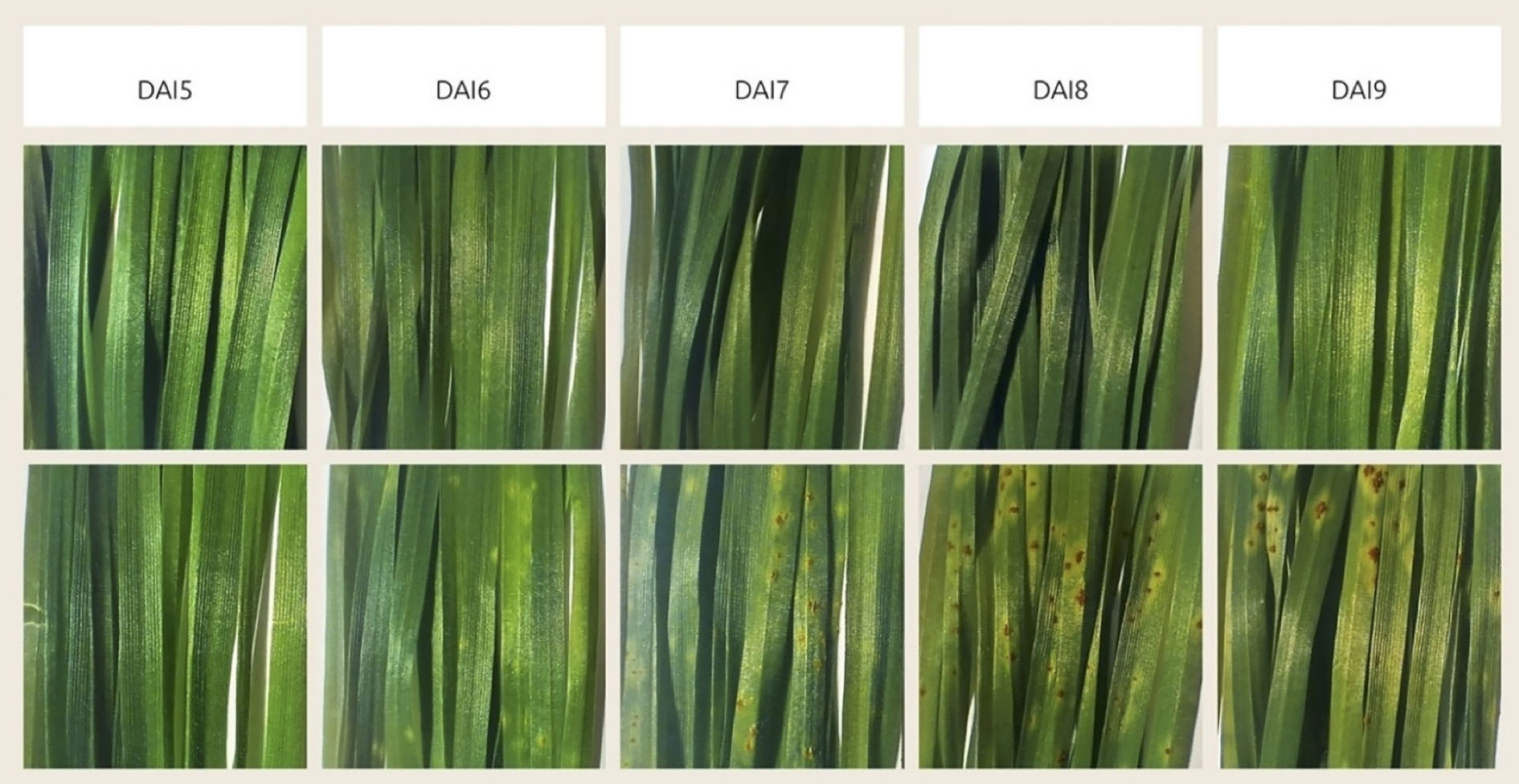
Disease progression visualization, showing the change in visible stem rust symptoms by days after inoculation (DAI). Top row demonstrates control group, bottom row demonstrates experimental group, inoculated with *Pgt*. The days preceding 5 DAI are not shown because there was no visible difference between them and 5 DAI.

When the urediniospores of the fungus get on the host plant leaf, they germinate with a germ tube through the stomata. They form in a mass under the epidermis of the affected plant organ - a leaf or a stem, and then break through it and appear as powdery elongated pustules - uredinia (Leonard and Szabo, 2005). The urediniospores of the fungus are formed 7–15 days after infection (Schumann and Leonard, 2000). As can be seen from **Figure 8**, in our experiment the stem rust pathogen development on the leaves of infected plants of the susceptible variety “Saratovskaya 74” proceeded as standard. The first noticeable chlorosis was visible on the surface of the leaves on the 6th day after infection, small pustules were visible to the naked eye on the 6-7th day and large pustules (infection type 3-4) on the 8th day after infection. On the 11th day after infection, these were developed pustules (infection type - 4).

### 3.2 Data analysis overall results

We conducted a statistical analysis of different hyperspectral data preprocessing strategies impact on classification model accuracy. Linear and nonlinear SVM models built on either the average hyperspectral data or their first derivatives were employed, as described in the Materials and Methods section. In analyzing the effect of each preprocessing strategy, the distribution of its test F1-score was constructed based on various scenarios. In each scenario, only the specific preprocessing method was applied, and its validation F1-score reached a maximum.

Among pixel-wise preprocessing methods, the minmax and standard normalization algorithms without Savitzky-Golay smoothing achieved the best results, with F1-scores ranging from 0.86 to 0.92. Applying additional smoothing with the Savitzky-Golay algorithm negatively impacted the accuracy of the average curve model in nearly all cases; however, this was not true for the combination of smoothing with first-derivative models. The standard normalization followed by Savitzky-Golay smoothing yielded the best results, with F1-scores between 0.91 and 0.96. Robust normalization performed worse than other standardization algorithms, both without smoothing (F1-score of 0.75–0.85) and with smoothing (0.68–0.92).

Among curve-wise normalizations, the standard algorithm achieved the best results, with an F1-score of 0.79–0.88 for the average model and 0.89–0.90 for the first-derivative model. Not applying any preprocessing also provided good results, with F1-scores of 0.83–0.84 for the average model and 0.83–0.89 for the first-derivative model. The worst results for the average model were achieved with robust normalization, yielding an F1-score of 0.67–0.84, while for the first-derivative model, minmax normalization performed with an F1-score of 0.55–0.67.

For channel-wise preprocessing, the standard normalization method showed a notable advantage, with F1-scores of 0.83–0.86 compared to 0.68–0.90 for minmax, 0.56–0.87 for robust, and 0.66–0.92 for no channel-wise standardization applied. Although other standardization methods reached higher maximum F1-scores than standard normalization, their F1-score distributions had greater variance and lower minimum values.

Regarding grouping strategies for channel-wise normalization, day-based grouping significantly outperformed other strategies, with an F1-score distribution of 0.77–0.87 compared to 0.5–0.84 for experiment-based grouping and 0.67–0.84 for applying channel-wise standardization across the entire dataset. This indicates that day-based grouping, based on the hypothesis that external noise sources vary daily, provides the best strategy; thus, statistical parameters for each day’s sample should be standardized independently.

A day-based grouping analysis of model accuracy distribution revealed that the first-derivative model significantly outperformed the average model, especially on the 4th day post-inoculation, with F1-scores of 0.62–0.83 compared to 0.54–0.57. On day 7, the average curve model outperformed the first-derivative model, with F1-score distributions of 0.93–0.96 compared to 0.84–0.89. On days 5, 6, and 8, the first-derivative model outperformed the average curve model and showed substantially lower F1-score variance.

Feature importance analysis showed that:

- The model based on absolute values relied more heavily on the green spectrum range than the derivative model, with an importance score of 0.4 versus 0.3.
- Over 25% of feature importance for both models were in the red spectrum range - 0.26 for the average curve model and 0.38 for the first-derivative model.
- The first-derivative model relied on the infrared spectrum range significantly more than the average curve model, with importance scores in the infrared range of 0.21 for the first-derivative model and 0.004 for the average model.

**Figure 9:**
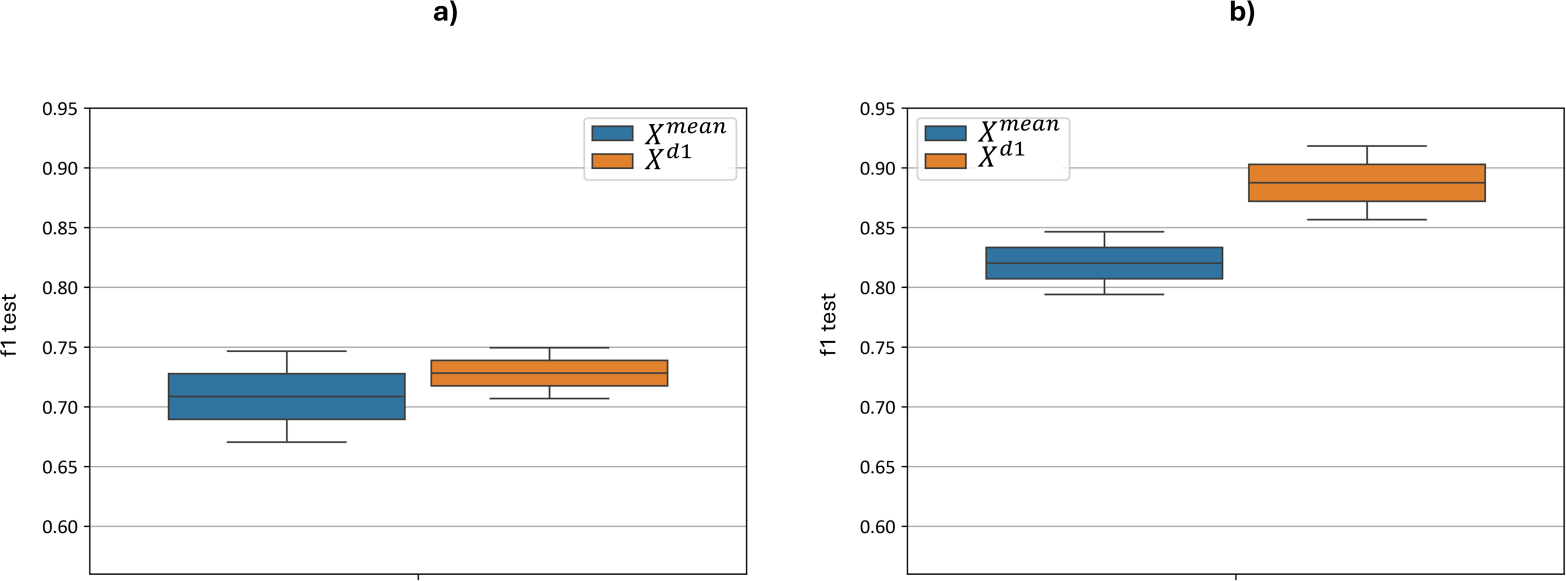
Test F1-metric distribution for each feature set $X^{mean}$ and $X^{d1}$ models, based on the best cross-validation metric. “f1 test” denotes the *F1* values. **(a)** demonstrates F1 scores with no preprocessing, **(b)** demonstrates F1 scores after applied preprocessing.

**Figure 9** illustrates the distribution of the test F1-metric for each feature set based on the best cross-validation metric. Without preprocessing, the F1 score ranges from 0.67 to 0.75 for the $X^{mean}$ model and from 0.71 to 0.75 for the $X^{d1}$ model. When optimizing with additional preprocessing, the F1 score ranges were improved from 0.79 to 0.85 for the $X^{mean}$ model and from 0.86 to 0.92 for the $X^{d1}$ model. The implementation of additional preprocessing led to a statistically significant improvement: for $X^{mean}\;$paired t-test yielded a *p-value* = 5.6 ×10–^9^ and for $X^{d1}$ paired t-test yielded *p-value* = 5.7 ×10^-8^. Thus, applying the proposed sequence of hyperspectral data preprocessing steps prior to classification leads to a substantial improvement in overall classification performance.

### 3.3 Preprocessing comparison

This section presents the results of evaluating various preprocessing approaches, including pixel-wise, curve-wise, and channel-wise normalization, as well as the application of the Savitzky–Golay smoothing algorithm. The following figures show the distributions of performance metrics for the best-performing models using these methods.

**Figure 10:**
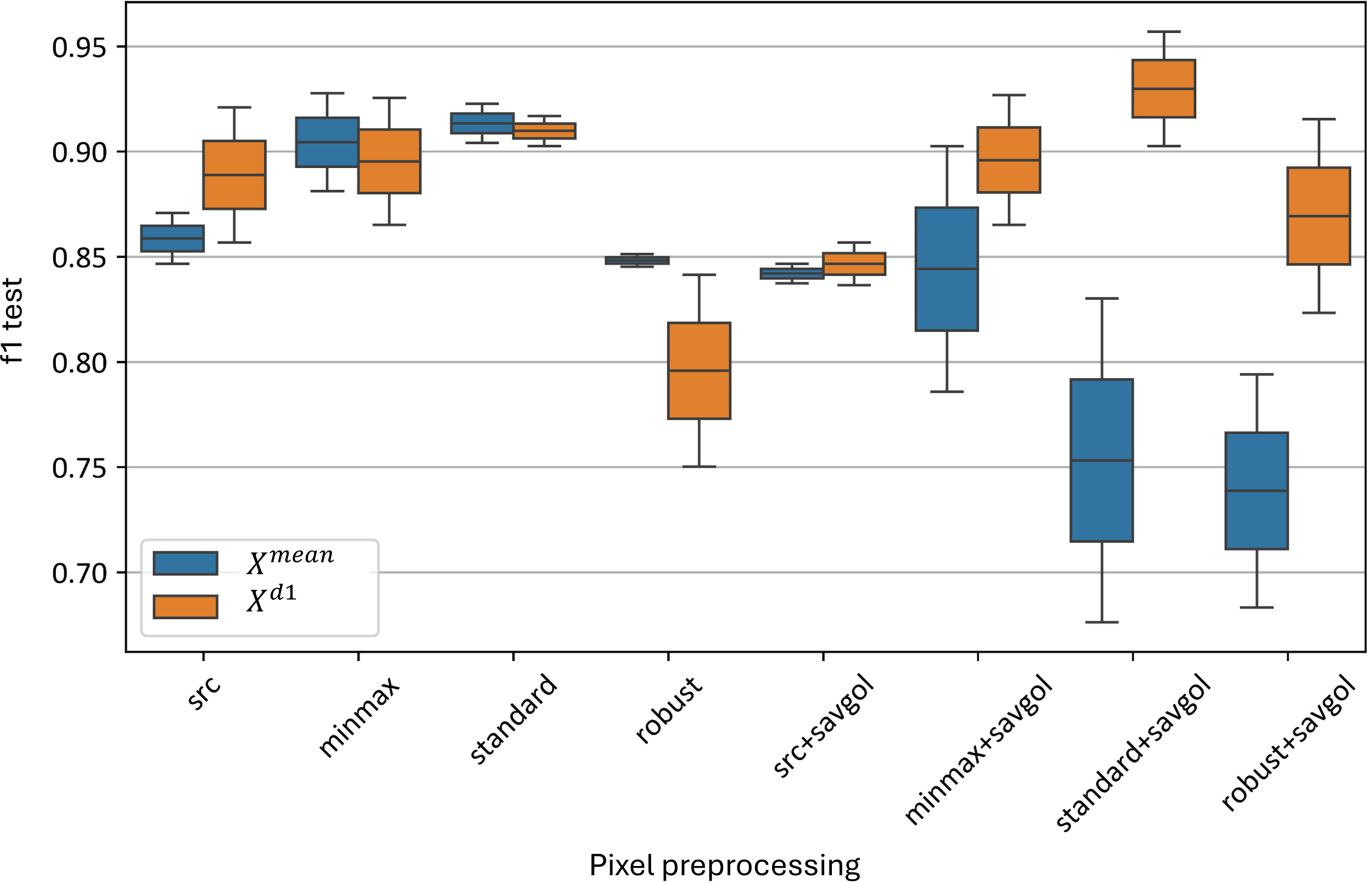
Test F1-metric distribution for top $X^{mean}$ and $X^{d1}$ models, using pixel-wise preprocessing methods. “f1 test” denotes the *F1* values. “src” denotes the data without preprocessing, “minmax”, “standard” and “robust” are corresponding to the normalization methods, and “savgol” denotes the application of Savitzky-Golay smoothing, following the normalization.

**Figure 10** demonstrates the impact of various pixel-wise preprocessing methods on the test metrics of the best-performing models. On average, across all models, the minmax and standard normalizations without Savitzky-Golay smoothing provided the best results, with F1-scores ranging from 0.86 to 0.92. Applying additional Savitzky-Golay smoothing generally decreased the accuracy of the $X^{mean}\;$model in almost all cases; however, this effect was not observed for combinations involving smoothing and the $X^{d1}\;$model. The standard normalization followed by Savitzky-Golay smoothing yielded the highest F1-scores, between 0.91 and 0.96. The standard normalization without Savitzky-Golay also yielded comparable F1-scores, between 0.91 and 0.92. Robust normalization performed worse than other standardization algorithms, both with (F1-score of 0.68–0.92) and without smoothing (F1-score of 0.75–0.85).

**Figure 11:**
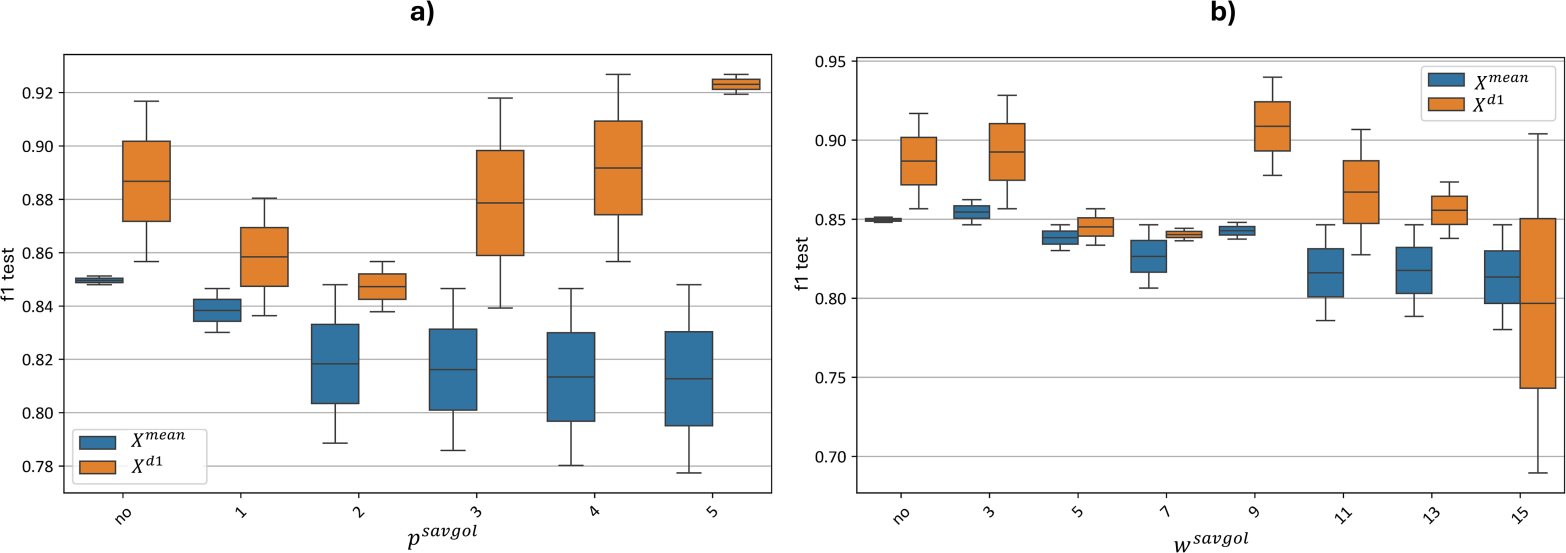
Test metrics distribution for top $X^{mean}$ and $X^{d1}$ models with varying Savitzky-Golay smoothing parameters. “f1 test” denotes the F1 values. The figure illustrates changes in the F1-score distribution of the best models as parameters of the Savitzky-Golay smoothing algorithm are varied. .

**Figure 11a** illustrates the influence of the smoothing polynomial *p^savgol^* degree. **Figure 11b** illustrates the influence of the smoothing polynomial *w^savgol^* window width.

**Figure 11** illustrates changes in the F1-score distribution of the best models as parameters of the Savitzky-Golay smoothing algorithm were varied: (a) the degree of the smoothing polynomial *p^savgol^* and (b) the window width of the smoothing polynomial *w^savgol^*.

**Figure 11a** shows that the accuracy of models built on $X^{mean}$ features declined as the polynomial degree increased, decreasing from 0.84–0.85 without smoothing to 0.78–0.85 at the maximum polynomial degree of 5. In contrast, the $X^{d1}\;$model exhibited a shift in F1-score distribution toward higher values as the polynomial degree increased, reaching a distribution of 0.92–0.93 at the maximum polynomial degree of 5.

**Figure 11b** similarly shows a drop in accuracy for the $X^{mean}\;$model as the window width increased, from 0.84–0.85 without a smoothing window to 0.78–0.85 at the maximum window width of 15.

**Figure 12:**
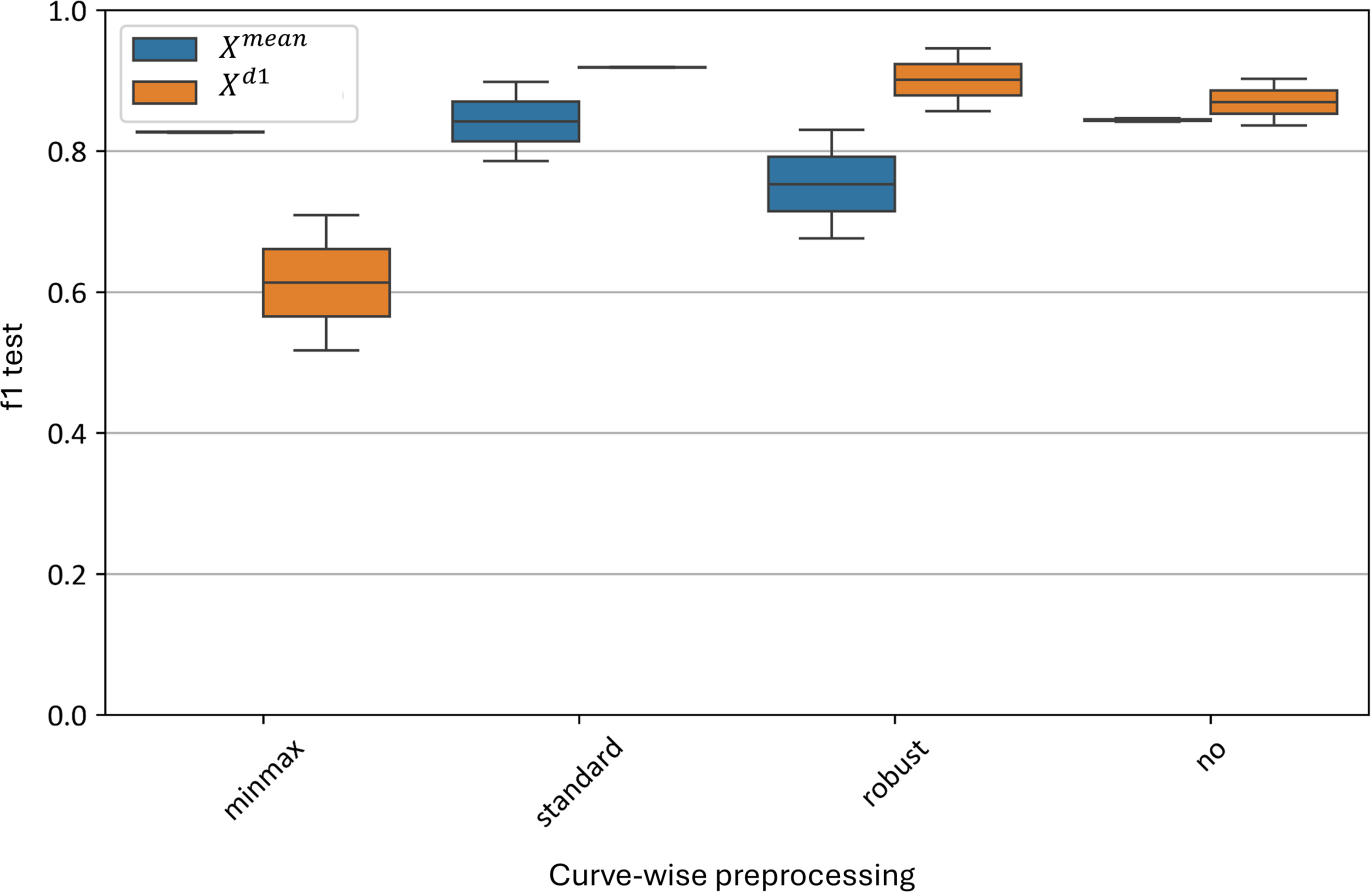
Test metrics distribution for top $X^{mean}$ and $X^{d1}$ models using different curve-wise normalization algorithms. “f1 test” denotes the *F1* values. “minmax”, “standard” and “robust” are corresponding to the normalization methods and “no” denotes data without any preprocessing.

**Figure 12** illustrates the impact of various curve-wise preprocessing methods on the test metrics of the best models. The standard normalization algorithm yields the best results, with F1-scores ranging from 0.79 to 0.88 for the $X^{mean}\;$ model and 0.89 to 0.90 for the $X^{d1}$ model. Similarly, good results are observed without any preprocessing, with F1-scores of 0.83–0.84 for the $X^{mean}$ model and 0.83–0.89 for the $X^{d1}\;$model.

The worst result for the average curve model ( $X^{mean}$) is with robust normalization, which yields an F1-score of 0.67–0.84. For the $X^{d1}$ model, the minmax normalization performs the worst, with an F1-score range of 0.55–0.67.

Thus, at the curve-wise level, the standard preprocessing method proves to be the most effective for the averaged spectral curves.

**Figure 13:**
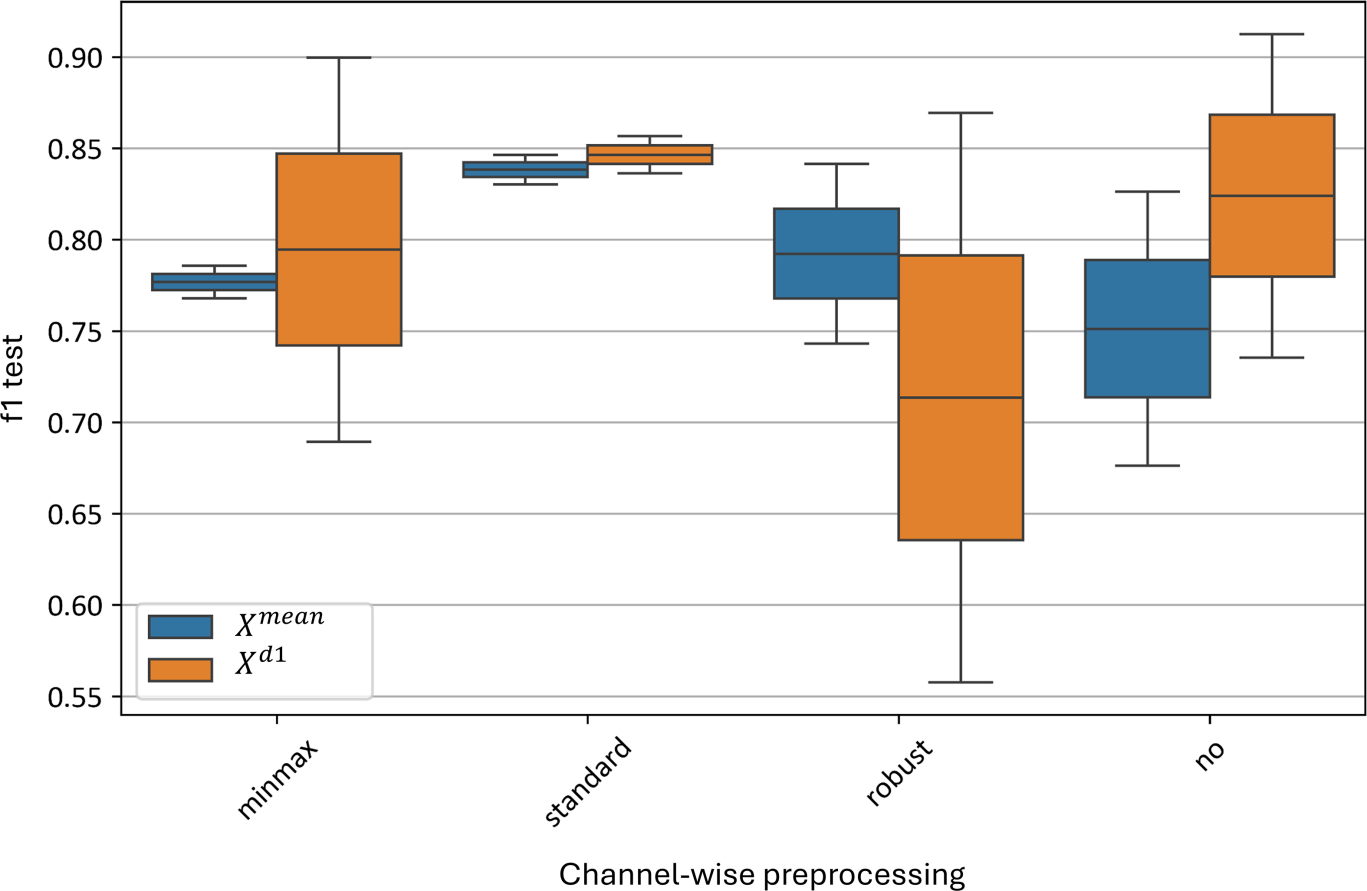
Test metrics distribution for top $X^{mean}$ and $X^{d1}$ models using different channel-wise normalization algorithms for variance equalization. “f1 test” denotes the *F1* values. “minmax”, “standard” and “robust” are corresponding to the normalization methods and “no” denotes data without any preprocessing.

**Figure 13** illustrates the impact of various channel-wise preprocessing methods on the test metrics of the best models. The standard normalization method at this level significantly outperforms the others, with F1-scores ranging from 0.83 to 0.86, compared to 0.68–0.90 for the minmax method, 0.56–0.87 for robust normalization, and 0.66–0.92 when no channel-wise standardization is applied. Although the maximum F1 values of other standardization methods exceed those of the standard method, their distributions show considerably greater variance and lower minimum F1 scores.

Thus, at the channel-wise level, the standard preprocessing method also proves to be the most effective for the averaged spectral curves, similarly to the curve-wise level.

**Figure 14:**
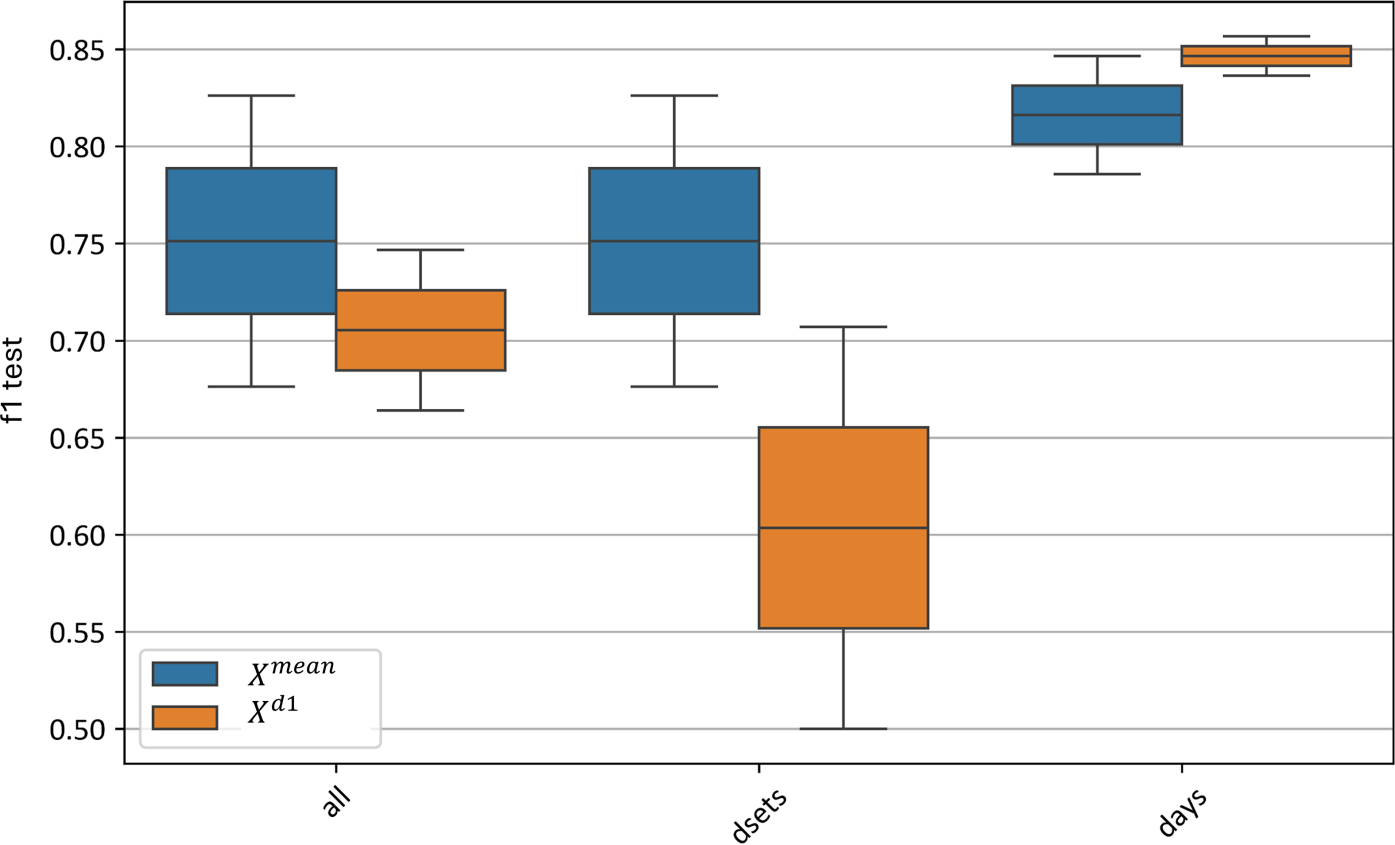
Test metrics distribution for top $X^{mean}$ and $X^{d1}$ models using different grouping strategies in channel-wise normalization for variance equalization. “f1 test” denotes the *F1* values. “all” corresponds to no grouping applied, “dsets” corresponds to experiment-based grouping and “days” corresponds to day-based grouping.

**Figure 14** demonstrates the effect of different grouping strategies in channel-wise preprocessing on the test metrics of the top models. The F1-score distribution with day-based grouping significantly outperforms other strategies, with values ranging from 0.77 to 0.87 for day-based grouping, 0.5 to 0.84 for experiment-based grouping, and 0.67 to 0.84 when no grouping is applied, using channel-wise standardization on the entire dataset. This analysis suggests that day-based grouping is the most effective data grouping strategy, supporting the hypothesis that external noise sources vary substantially from day to day. As a result, the statistical parameters of each day’s sample should be standardized independently from others.

**Figure 15:**
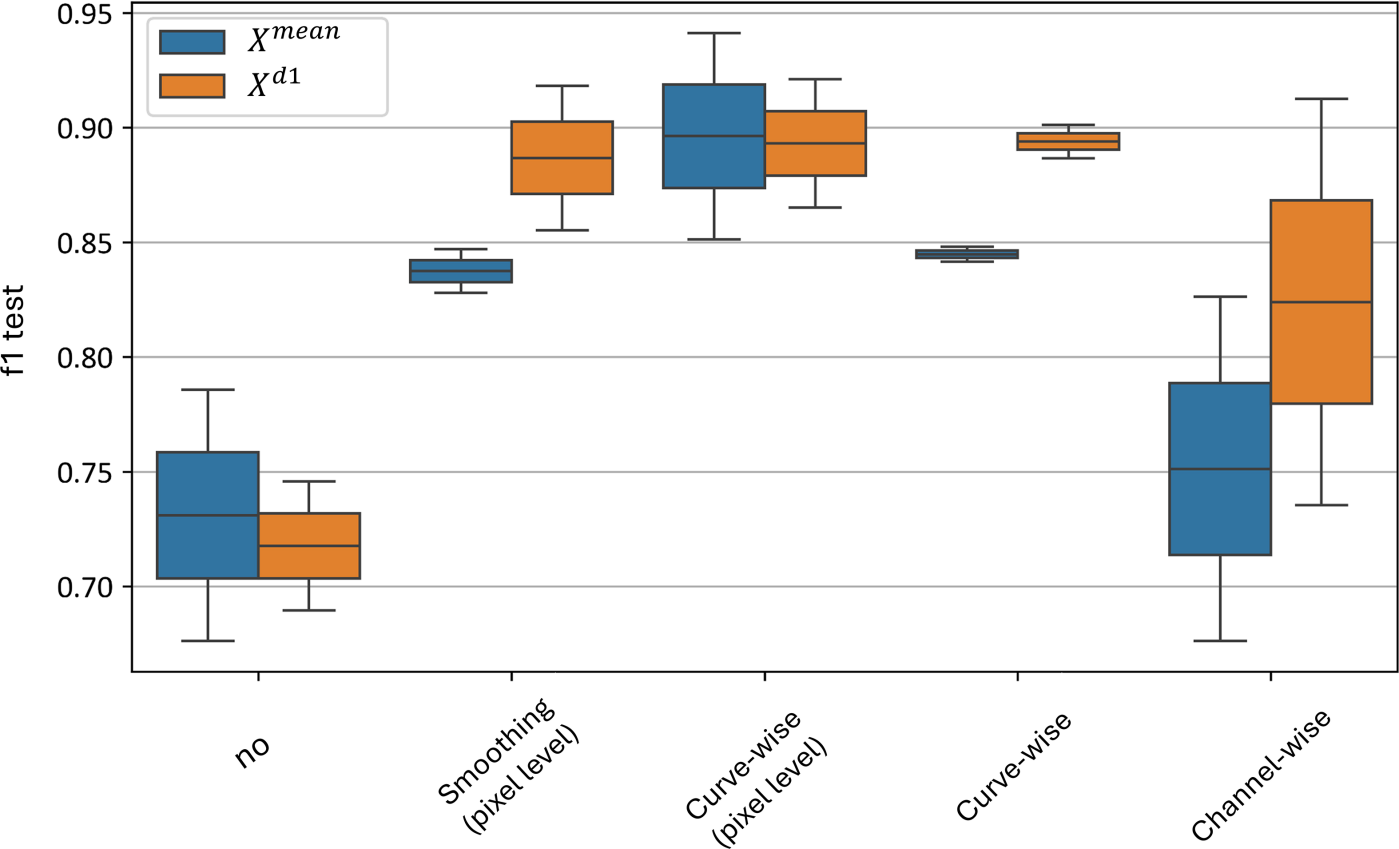
Test metrics for top $X^{mean}$ and $X^{d1}$ models’ distribution for independent application of preprocessing techniques. “f1 test” denotes the *F1* values. “No” corresponds to no preprocessing applied, other values denote the corresponding preprocessing methods.

**Figure 15** illustrates the distributions of the F1-score when each preprocessing level is independently applied, excluding all other levels during each application. The figure shows that, despite high classification accuracy achieved when the channel-wise preprocessing is used in combination with other preprocessing techniques, its performance drops significantly when curve-wise preprocessing is absent - with an F1-score distribution of 0.68–0.82 for the $X^{mean}$ model and 0.74–0.92 for the $X^{d1}$ model.

The best results are achieved with curve-wise preprocessing at the level of pixel hyperspectral curves, yielding an F1-score distribution of 0.85–0.94 for the $X^{mean}\;$model and 0.87–0.92 for the $X^{d1}$ model. Additionally, curve-wise preprocessing applied to the averaged curves across all plant pixels demonstrates high mean F1-scores with the lowest variance - an F1-score distribution of 0.84–0.85 for the $X^{mean}$ model and 0.88–0.90 for the $X^{d1}$ model.

Thus, the curve-wise preprocessing family demonstrates its superiority in independent application at both the pixel and averaged-curve levels, and it can be effectively used to build an accurate classifier without relying on other preprocessing techniques.

### 3.4 Machine learning-models comparison

The following machine learning algorithms were used to analyze the preprocessed data.

Support vector machine (SVM) (Cortes and Vapnik, 1995) is a powerful supervised learning model primarily used for classification. At its core, the linear SVM algorithm aims to find the optimal separating hyperplane that maximizes the margin between the two classes. The margin is defined as the distance from the hyperplane to the closest data points from each class, which are called support vectors. This approach of maximizing the margin often leads to better generalization on new data compared to classifiers like Logistic Regression, which only minimize the empirical error without explicitly maximizing the margin.

A key strength of SVM is its ability to perform nonlinear classification using the kernel trick. Instead of explicitly mapping the data into a high-dimensional feature space (which can be computationally expensive), the kernel trick allows the algorithm to operate by computing similarity functions (kernels), such as the Radial Basis Function (RBF) or polynomial kernels, between all pairs of original data points. This effectively enables SVM to construct complex nonlinear decision boundaries while retaining the computational efficiency of a linear model. As tuning hyperparameters of model we use different regularization values and different kernels. A more detailed description of hyperparameters is provided in **Table 4**.

Logistic regression (LR) (Hosmer and Lemeshow, 2000) is a linear classification model. As a tuning hyperparameters of model we used different solvers and regularizations including L1 and L2 norms with different weights. A more detailed description of the hyperparameters is provided in **Table 4**.

Light Gradient Boosting Machine (LGBM) (Ke et al., 2017) is an extension of gradient boosting algorithm (Friedman, 2002) designed to reduce time consumption when building the ensemble of decision trees. For this purpose, Gradient-based One-Side Sampling (GOSS) and Exclusive Feature Bundling (EFB) techniques are used. The idea of GOSS is to exclude data instances with small gradients on each step of gradient optimization, while EFB tries to bundle mutually exclusive features to reduce the number of features without impairing accuracy. As tuning hyperparameters of model we use number of estimators, maximum tree depth, learning rate, L1 and L2 regularizations. A more detailed description of hyperparameters is provided in **Table 4**.

The selection of models in the set was based on the following criteria. We used SVM algorithm which has already proven its applicability for the hyperspectral data classification and has been successfully applied in (Baek et al., 2019; Guo et al., 2020). We used Logistic Regression because in our same previous work (Terentev et al., 2023) it has been shown that linear SVM sometimes demonstrates even better test performance than its nonlinear implementation and that is a reason to keep explore applicability of different linear models to hyperspectral data classification problem. Also, we used LGBM model to explore nonlinear classifiers applicability to our problem.

**Figure 16:**
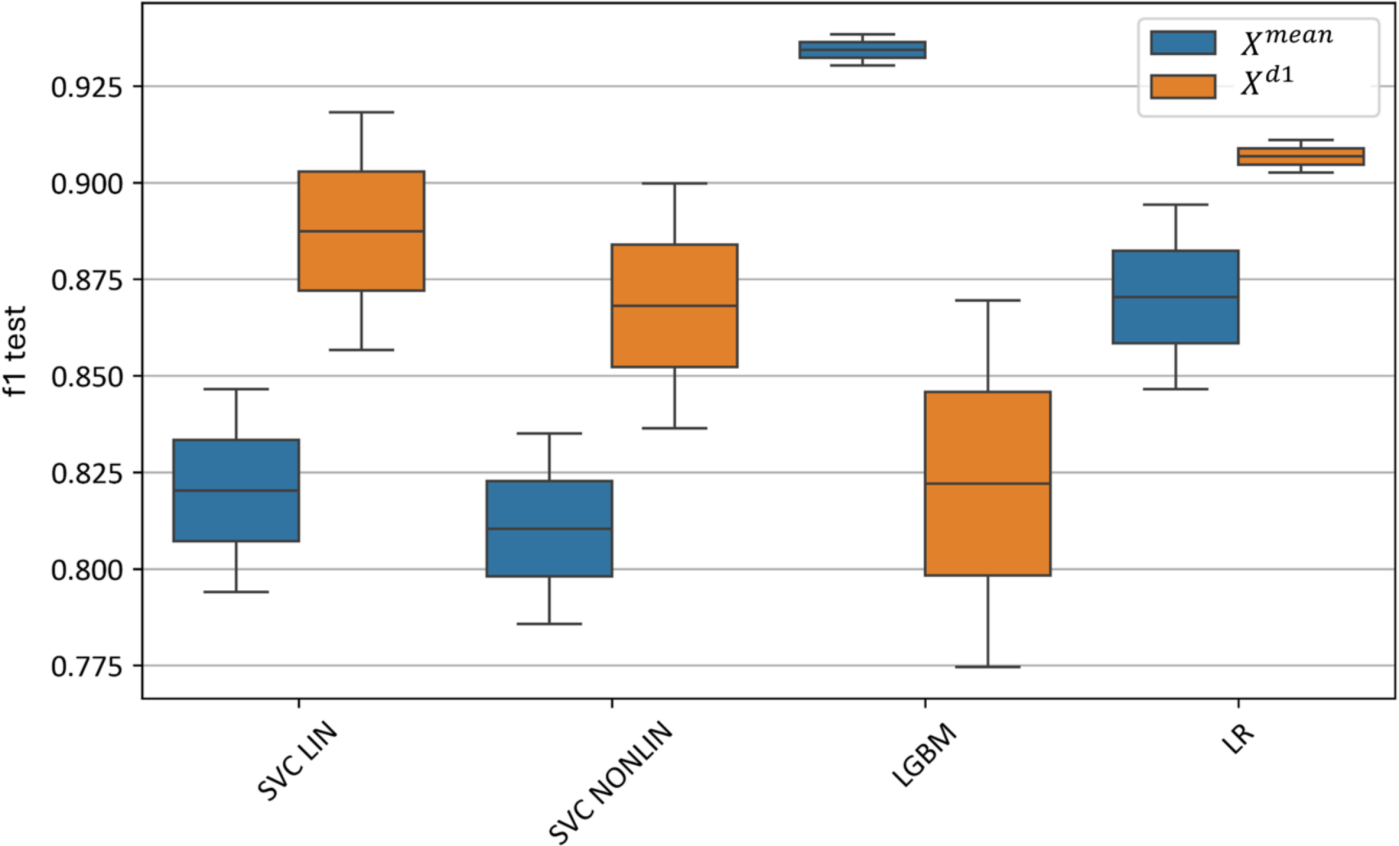
Test metrics for top $X^{mean}$ and $X^{d1}$ models using different machine learning algorithms. “f1 test” denotes the F1 values. “SVC LIN” and “SVC NONLIN” correspond to best linear and non-linear SVM models, respectively. “LGBM” and “LR” correspond to best Light-Gradient boosting and Logistic Regression models, respectively.

**Figure 16** illustrates the effect of selecting various machine learning models on test metrics. The best results for $X^{mean}$ are achieved by the LGBM model, with an F1-score of 0.93–0.94, while for $X^{d1}$, logistic regression performs the best with an F1-score of 0.90–0.91.

The linear SVM model slightly outperforms the nonlinear SVM model, with an F1-score of 0.79–0.84 for the linear SVM on $X^{mean}$ and 0.85–0.92 on $X^{d1}$. In comparison, the nonlinear SVM achieves an F1-score of 0.78–0.83 for $X^{mean}$ and 0.83–0.90 for $X^{d1}$.

Thus, using the feature-extraction algorithm based on the first derivatives of the hyperspectral curves ( $X^{d1}$) enables the construction of a linear model with the highest classification accuracy, whereas boosting achieves superior performance on the averaged curves ( $X^{\text{mean}}$) without additional feature extraction.

### 3.5 Error distribution analysis

This section examines the distribution of classification errors across different feature spaces and models to identify systematic misclassification patterns and potential data quality issues.

**Figure 17:**
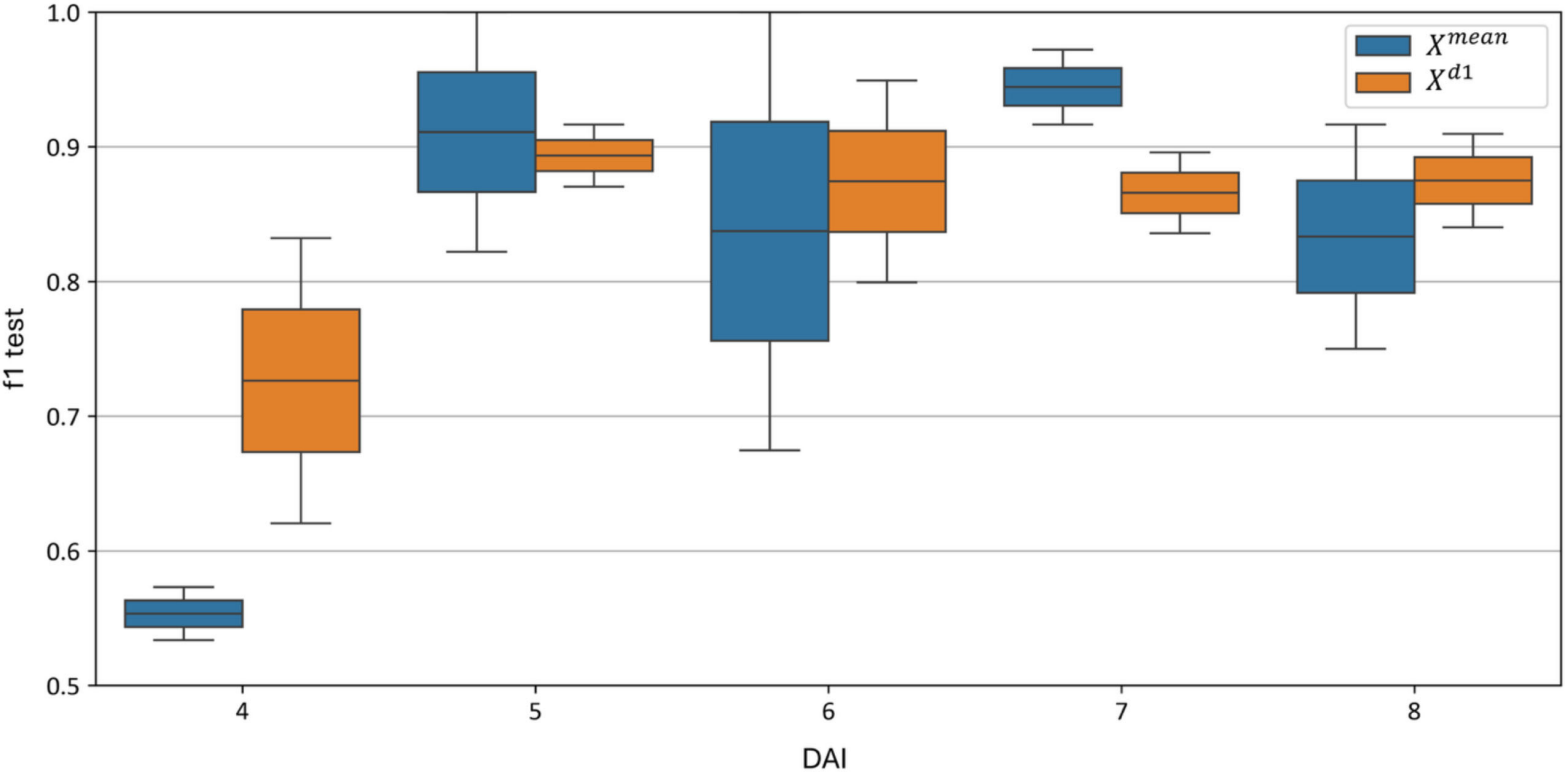
Test metrics for top $X^{mean}$ and $X^{d1}$ models’ dependence of test accuracy of various models by day. “f1 test” denotes the F1 values. The horizontal scale shows the days after inoculation (DAI).

**Figure 17** reveals a compelling trend. On average, the model using $X^{d1}$ features outperforms the model based on original curves ( $X^{mean}$). Notably, the F1-score of the $X^{d1}$ model significantly exceeds that of the $X^{mean}$ model on the earliest, 4th day of the experiment - 0.62–0.83 for $X^{d1}$ versus 0.54–0.57 for $X^{mean}$. On the 7th day, however, the F1-score distribution of the $X^{mean}$ model surpasses that of $X^{d1}$, with ranges of 0.93–0.96 and 0.84–0.89, respectively. On the remaining days (5, 6, and 8), the $X^{d1}$ model again outperforms the $X^{mean}$ model and shows a markedly lower F1-score variance ( $X^{mean}$ F1-scores are 0.82-1.00, 0.68-1.00, 0.75-0.92 and $X^{d1}$ F1-scores are 0.89-0.91, 0.80-0.95, 0.85-0.92 respectively).

During the error analysis stage, a subset of images was identified on which all top models (LGBM based on the $X^{mean}$ feature space and LR based on the $X^{d1}$) feature space) made errors, consistently misclassifying all control images as experimental. Further analysis revealed that problematic images could be filtered out using a threshold-based correlation filtering method, with a correlation threshold of 0.85 applied against the mean.

Formally, filtering by correlation with the mean can be defined as follows:

$x^{meanavg}=\{\frac{1}{864}\sum_{s=1}^{864}x_{s,i}^{mean}\;|\;i\in 1..106\;$}

$x^{d1avg}=\{\frac{1}{864}\sum_{s=1}^{864}x_{s,i}^{d1}\;|\;i\in 1..106\;$}

$X^{rmean}=\{x_{s,c}^{mean}:r(x^{d1avg},x_{s}^{mean})>0.85\}$

$X^{rd1}=\{x_{s,c}^{d1}:r(x^{meanavg},x_{s}^{d1})>0.85\}$

Where $x^{meanavg}$ is the mean curve calculated across the columns of matrix $X^{mean}$ represented as a 106-dimensional vector; $x^{d1avg}$ is the mean curve across the columns of matrix $X^{d1}$ represented as a 106-dimensional vector., $X^{rmean}$ and $X^{rd1}\;$are matrices derived from $X^{mean}$ and $X^{d1}$ respectively, by threshold-based row filtering. The function r denotes Pearson’s correlation, defined as:


$$r(x,y)=\;\frac{\sum_{x_{i}\in \;x}(x_{i}-mean(x))(y_{i}-mean(y))}{\sum_{x_{i}\in \;x}{\left(x_{i}-mean(x)\right)}^{2}\sum_{y_{i}\in \;y}{\left(y_{i}-mean(y)\right)}^{2}}$$

**Figure 18:**
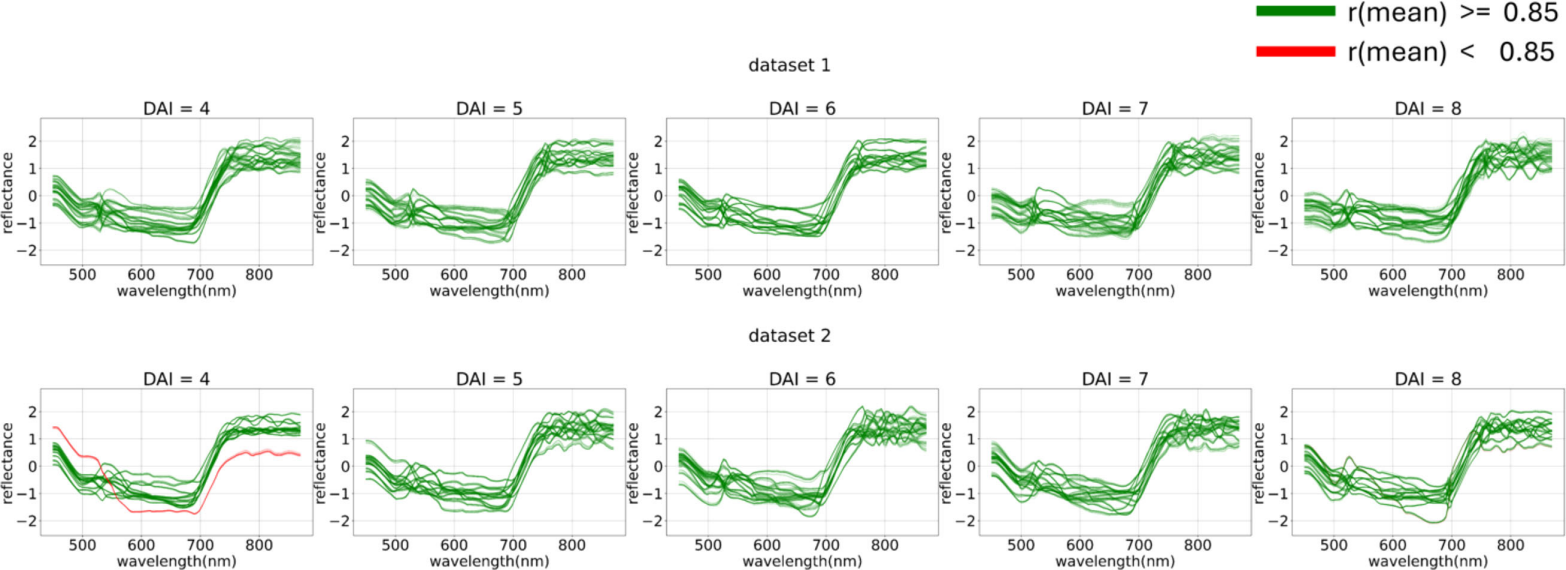
Filtering out-of-distribution curves via mean curve correlation. The curves that exceed (green) or fall short (red) with a correlation threshold of 0.85 applied against the mean are displayed. From 4 (DAI = 4) to 8 (DAI = 8) days after inoculation across both imaging sessions (top – first repetition of the experiment, bottom – second repetition of the experiment).

The filtered problematic curves are shown in **Figure 18**.

This filtering process effectively classified a portion of the errors as out-of-distribution data, thereby removing them from the main dataset.

### 3.6 Bias distribution sensitivity analysis

**Figure 19:**
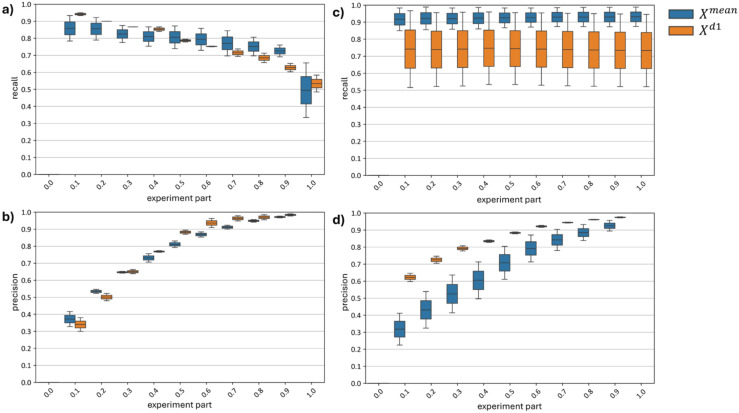
Changes in top models recall and precision metrics **(a, b)** with Channel-Wise Preprocessing and **(c, d)** Without Channel-Wise Preprocessing.

Since, unlike curve-wise preprocessing (a function of a single hyperspectral curve), channel-wise preprocessing depends on the distribution of the subset of hyperspectral curves, shifts in the proportion of diseased versus healthy plants within the normalized subset can lead to distribution bias post-normalization. To analyze the effect of distribution bias on model accuracy, curves showing the dynamics of recall and precision metrics as the proportion of diseased plants in the test set increases from 0 to 1 were constructed (**Figure 19**).

Subfigures (a) and (b) of **Figure 19** reveal a drop in recall (a) and a rise in precision (b) as the proportion of diseased plants in the test set increases. With models built using channel-wise normalization on a test set containing only diseased plants, about half of the diseased plants are misclassified as healthy. Precision reaches its maximum, meaning that all plants classified as diseased are indeed diseased.

Subfigures (c) and (d) of **Figure 19** show no change in recall (c) and an increase in precision (d) for models built without channel-wise normalization as the number of diseased plants in the test set grows. The $X^{mean}$ model demonstrates a robust recall range of 0.85–0.98, but its precision consistently falls short of that of the $X^{d1}$ model. When tested on a dataset with only 10% diseased plants, the $X^{mean}$ model achieves a precision of 0.21–0.42, indicating many false positives, while the $X^{d1}$model reaches a precision of 0.60–0.65 for the same test set.

**Figure 19** thus demonstrates that, although models utilizing channel-wise preprocessing generally achieve higher F1-scores under the current distribution of diseased and healthy plants (equal in our test set), testing on skewed distributions (where diseased or healthy plants dominate) shows these models are more sensitive to changes in the proportion of diseased and healthy plants in the test data. This sensitivity could be addressed by adjusting standardization based on prior information about the proportion of diseased plants relative to healthy ones; however, such information is not always available. Models using only curve-wise normalization are more robust to variations in the distribution of diseased and healthy plants in the test set, making them suitable for cases without additional prior information on disease prevalence or for single-image classification (since curve-wise normalization depends on a single curve, whereas channel-wise normalization depends on multiple curves).

### 3.7 ANOVA Statistic by preprocessing pipeline stages

**Figure 20:**
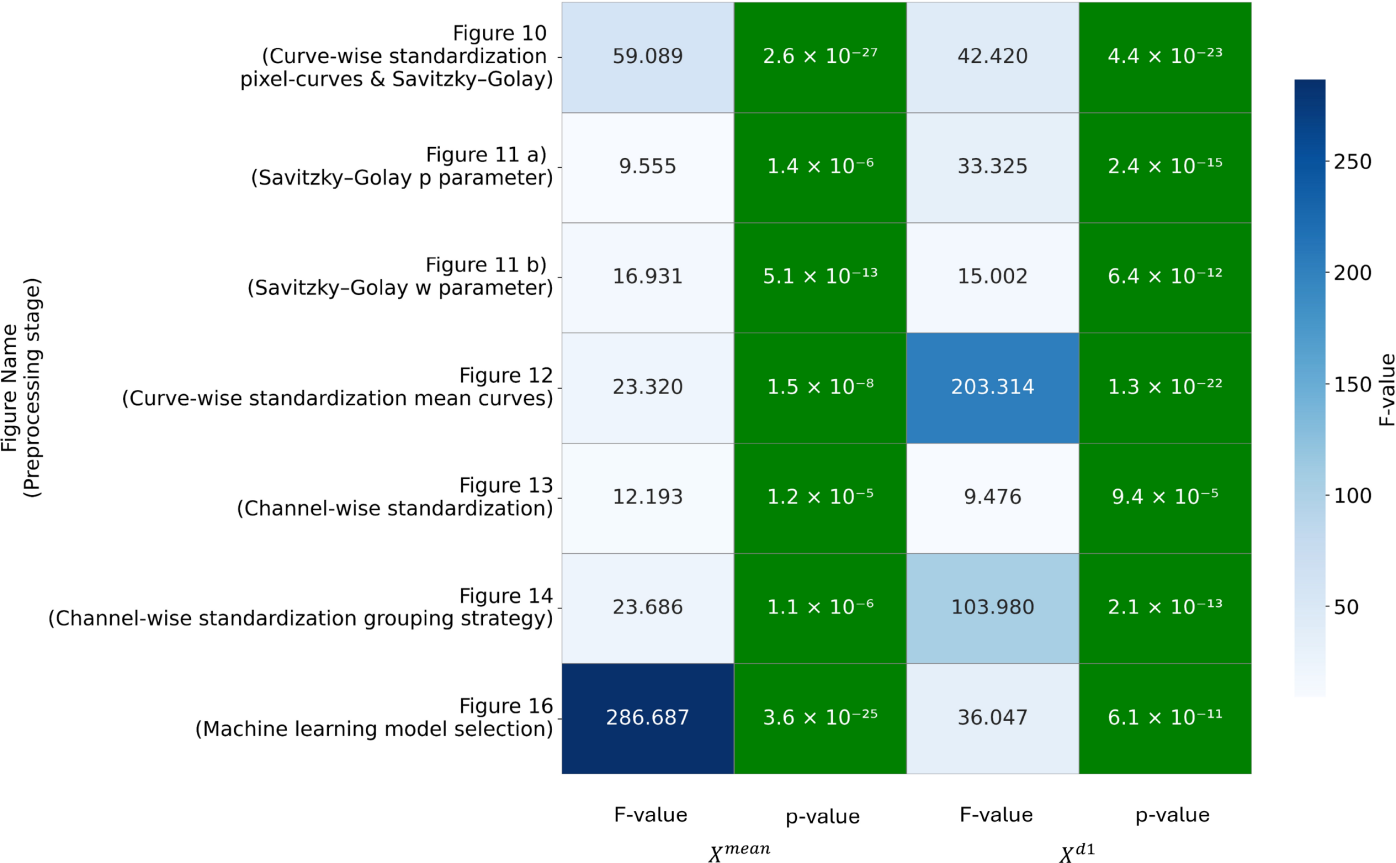
ANOVA statistics by preprocessing pipeline stages.

For each group comparison of the test F1-score distributions obtained using a given preprocessing option at each pipeline level, we computed the f-statistic and corresponding p-value using one-way ANOVA (St and Wold, 1989; McDonald, 2014) and its implementation from (Virtanen et al., 2020). **Figure 20** summarizes the ANOVA statistics for all preprocessing levels. All group comparisons exhibit extremely low false-positive probabilities, with p-values consistently below 10^−4^. The distributions of F-statistics differ markedly between the $X^{mean}\;$ and $X^{d1}$ feature spaces.

On **Figure 20** the *F-value* represents the ratio of between-group variance to within-group variance; higher values (darker blue) indicate a stronger effect of the preprocessing choice at that stage. The *p-value* denotes the probability of observing differences when no true differences exist (lighter shading corresponds to higher probability). The y-axis indicates the preprocessing stage within the overall pipeline, with reference to the previously described figure showing the statistical comparison of test-metric distributions, accompanied by a brief textual description of each preprocessing step.

For the $X^{mean}\;$ feature space, the highest F-statistic is observed at the stage corresponding to the choice of machine-learning model. This indicates that, when working with $X^{mean}$, model selection is the dominant factor influencing performance.

In contrast, for the $X^{d1}$ feature space, the highest F-statistics occur at the curve-wise standardization step and at the stages related to data grouping strategies for channel-wise standardization. This suggests that, when operating in $X^{d1}$, curve-wise and channel-wise preprocessing steps are critical. Appropriate curve-wise normalization and well-defined channel-wise grouping strategies can effectively compensate for the limited generalization capacity of linear models relative to boosting methods and can enable the construction of simpler yet more robust solutions - for example, in early-infection scenarios (**Figure 17**).

### 3.8 Model importance analysis

After training, we conducted feature importance analysis on each of the best classification models. To interpret the models, we estimated the normalized importance of individual features (wavelengths) based on the internal structure of each trained algorithm.

In the logistic regression model, the predicted logit is a linear combination of the input hyperspectral values. Therefore, the absolute value of the *i*-th coefficient directly reflects the influence of the *i*-th wavelength on the model output prior to the sigmoid transformation. To obtain normalized absolute importances, we compute the absolute value of each coefficient and divide it by the sum of absolute values across all coefficients.

For the LGBM model, feature importance was assessed using the gain metric, which quantifies the total improvement in the objective function contributed by a feature across all tree nodes where it is used. The resulting importance values were then normalized in the same manner as for the linear model to ensure comparability.

Thus, for the $X^{mean}\;$ model, the importance vector lies in ∈ R^106^, and for the $X^{d1}$ model, the importance vector lies in ∈ R^105^. The elements of each importance vector are non-negative values defined on the interval [0, 1] and their sum equals 1.

The top model for average curves was LGBM, while for first derivatives, it was Logistic Regression. Linear models are inherently interpretable and do not require additional algorithms to explain their internal logic. For analyzing feature importance in the LGBM model, we used gain-based feature importance.

**Figure 21:**
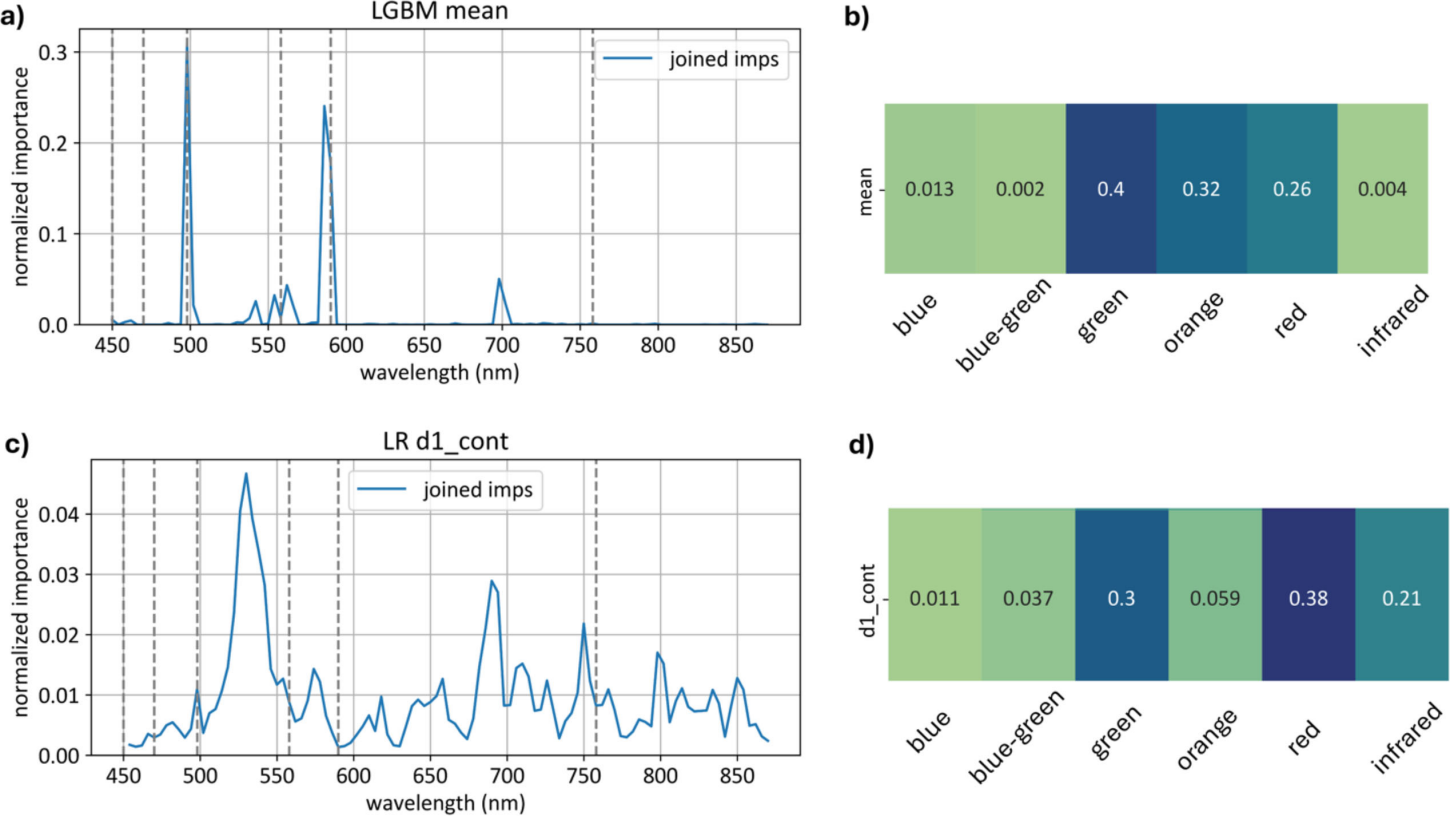
Top models normalized importances built on $X^{d1}\;$and $X^{d1}\;$features. Subfigure **(a)** Channel-wise importance shares of the top model built on $X^{mean}$ features. Subfigure **(b)** Importance aggregated across color ranges for the $X^{mean}\;$model. Subfigure **(c)** Channel-wise importance shares of the top model built on $X^{d1}\;$features. Subfigure **(d)** Importance aggregated across color ranges for the $X^{d1}\;$model.

**Figure 21** displays the importance of each wavelength in the models built on the basic feature sets (absolute curve values and first derivatives) and aggregate importance values across different spectral ranges.

The $X^{mean}$ model relies on the green range significantly more than the $X^{d1}$ model, with importance scores of 0.40 compared to 0.30.

The $X^{d1}$ model, on the other hand, relies heavily on the red range, with a total importance of 0.38 for first derivatives compared to 0.26 for the average curve model.

Additionally, the $X^{d1}$ model places substantially more emphasis on the infrared range, with an importance score of 0.210, whereas for $X^{mean}$, the infrared range importance is only 0.004.

These findings highlight distinct dependencies of each model on specific wavelength ranges, underscoring the green range’s prominence for models build on $X^{mean}$ feature space and the red and infrared ranges’ importance for models build on $X^{d1}$ feature space.

### 3.9 Key experimental findings

The results demonstrate that preprocessing significantly influences the accuracy and stability of hyperspectral data classification for early wheat stem rust detection. Curve-wise normalization and derivative-based feature extraction provided the most consistent improvements in F1-scores compared to raw spectra, particularly during early disease stages. Among channel-wise normalization strategies, day-based grouping yielded the highest performance, highlighting the importance of accounting for daily variability in plant conditions.

In terms of model comparison, Light Gradient Boosting Machine achieved the best overall performance on average curves, whereas Logistic Regression showed superior results on derivative features, with both outperforming SVM variants. Error distribution analysis revealed systematic misclassification patterns related to out-of-distribution samples, which were successfully mitigated using correlation-based filtering. Sensitivity analysis showed that models using channel-wise normalization were more vulnerable to class imbalance, while curve-wise-only normalization offered greater robustness under distribution shifts.

Finally, feature importance analysis indicated that average-curve models rely heavily on the green spectrum, whereas derivative-based models emphasize red and near-infrared wavelengths, reflecting their distinct sensitivity to physiological changes in plant tissues. These findings underscore the necessity of selecting appropriate preprocessing strategies and feature representations to enhance model accuracy and generalization in field conditions.

## 4 Discussion

Early detection of plant diseases is a critical challenge in precision agriculture and has been extensively discussed in the literature (Zhang et al., 2020; Shahi et al., 2023). Despite numerous efforts, no universally effective solution has been established (Bock et al., 2020; Bohnenkamp et al., 2021; Qi et al., 2021; Terentev et al., 2022). Our previous work demonstrated the feasibility of early disease detection using detached wheat leaves with hyperspectral imaging combined with an SVM classifier, achieving high accuracy from day 4 post-inoculation (Terentev et al., 2023). However, these conditions did not reflect real-world variability.

In this study, we addressed this gap by simulating field-like conditions through imaging intact potted plants at realistic planting densities. Unlike highly controlled experiments with uniform leaf positioning and stable illumination, this setup introduced challenges such as uneven lighting, leaf overlap, and edge pixels with mixed spectral signatures - factors frequently encountered in field imaging (Sankaran et al., 2011; Whetton et al., 2018; Abdulridha et al., 2020). These factors resulted in atypical reflectance patterns and increased the importance of a robust preprocessing pipeline.

The wheat sprouts were inoculated at the two-leaf stage (BBCH 12), and due to the growth characteristics of the pathogen, diseased and healthy leaves appeared intermixed within the same images. After urediniospore deposition, a local dikaryotic intercellular mycelium develops, forming individual uredinia, each originating from a single spore (Leonard and Szabo, 2005). First visible pustules typically appear 7–10 days after infection; therefore, leaves that emerged several days after inoculation remained uninfected by the time the experiment concluded. From a physiological standpoint, stem rust infection induces a series of interconnected changes in wheat plants, including reduced chlorophyll content, altered pigment composition, disturbed water balance, and elevated levels of free amino acids and soluble sugars (Bolton et al., 2008; Khaled et al., 2018; Ube et al., 2019). These alterations collectively modify the plant’s spectral signature: in the visible (VIS) region, reflectance decreases in the green and red wavelengths as chlorophyll breaks down, while in the near-infrared (NIR) region, reflectance declines due to damage to internal leaf structures and reduced cellular turgor (Mahlein, 2016; Jacquemoud and Ustin, 2019; Terentev and Dolzhenko, 2023). This infection pattern is typical for rust fungi under field conditions, where the pathogen progresses upward from lower leaves until reaching the flag leaf (Kolmer, 2013). As the flag leaf is crucial for grain filling, timely stem rust detection helps protect the flag leaf with a fungicide application that {-will preserve the yield preserves grain yield. This infection morphology required a more advanced preprocessing pipeline than the one in our previous study (Terentev et al., 2023).

Our findings confirm that preprocessing is the key determinant of classification performance under realistic conditions, which is supported by previous studies (Xu et al., 2020; Witteveen et al., 2022; Cozzolino et al., 2023). The baseline SVM model trained following the configuration from (Terentev et al., 2023) showed poor accuracy, which was expected given the additional noise sources. Subsequent analysis demonstrated that normalization and smoothing significantly improved classification metrics, increasing F1-scores from 0.67–0.75 to 0.86–0.94 across tested models. In particular, curve-wise preprocessing effectively mitigated illumination variability, while channel-wise normalization - especially using standard scaling - further enhanced stability and precision. This aligns with recent trends in the literature that emphasize how carefully designed preprocessing and feature extraction from hyperspectral data can close the gap between traditional statistical/shallow ML methods and more complex deep learning architectures (Paoletti et al., 2019; Helin et al., 2022).

An important observation is that the choice of scaling method strongly influences performance. Standard scaling consistently outperformed MinMax and Robust scaling at both curve-wise and channel-wise levels. This likely reflects the homogeneous distribution of reflectance values and the absence of strong outliers in the dataset. Interestingly, Robust scaling provided no advantage, reinforcing the conclusion that noise in this experiment was illumination-driven rather than anomaly-driven.

Feature representation also played a crucial role. Models trained on the first derivative of hyperspectral curves ( $X^{d1}$) systematically outperformed those based on raw mean reflectance ( $X^{mean}$), particularly for early detection. This suggests that derivative features capture subtle spectral changes preceding visible symptoms, a property especially relevant for asymptomatic classification (Zhang Y. et al., 2024). This robustness, especially for asymptomatic detection, was highlighted in other studies. For example, tomato bacterial leaf spot was detected days before visible symptoms by leveraging full-spectrum derivatives combined with ML classifiers (Zhang Y. et al., 2024). Such cross-domain parallels underscore the general utility of derivative-based feature spaces for early detection tasks.

Among the tested algorithms, gradient boosting (LGBM) and linear models (LR, linear SVM) delivered the highest F1-scores, with LGBM achieving 0.93–0.94 on $X^{mean}$ and LR achieving 0.90–0.91 on $X^{d1}$. Interestingly, the linear SVM slightly outperformed the nonlinear variant, likely due to the moderate dimensionality of the feature space and strong linear separability after preprocessing. Although deep models were not explored here, they remain a promising direction given the spectral–spatial nature of hyperspectral data. Importantly, the strong performance of simpler, more interpretable models demonstrates that properly preprocessed data {-may obviate-} {+may reduce+} the need for computationally expensive deep architectures - an observation echoed in several reviews (Hasan et al., 2019; Khan et al., 2022);.

Sensitivity analysis under class distribution shifts revealed that curve-wise preprocessing improves model robustness to imbalanced data, a key requirement for deployment in dynamic field conditions. Conversely, channel-wise preprocessing provided higher precision but was more sensitive to distribution changes and unsuitable for single-image inference, as it requires batch-level statistics. This observation is directly relevant to {-the field of-} domain adaptation and transfer learning, where robustness to distributional shift is one of the central challenges. Our preprocessing pipeline thus represents a transferable mathematical toolkit for tackling imbalance-driven bias in high-dimensional data analysis.

The wavelength importance analysis confirmed that derivative-based models rely more heavily on red and near-infrared bands, while raw reflectance-based models emphasize green wavelengths. This aligns with physiological indicators of stress and supports previous findings on vegetation spectral responses (Bruning et al., 2020; Terentev et al., 2022; Zhang Z. et al., 2024). Beyond the agricultural context, the mathematical tools we developed - curve-wise normalization, smoothing, and derivative-based features - represent a generalizable framework applicable to other hyperspectral and high-dimensional signals. Similar frameworks have already been successfully applied in soil nutrient mapping (Rossel et al., 2017), biomedical imaging (Liu et al., 2023), and environmental monitoring (Stuart et al., 2019), where noise reduction, distribution bias correction, and feature interpretability are critical.

A major constraint of the present work is that all experiments were carried out under controlled or semi-controlled laboratory settings, which do not fully capture the variability typical of open-field hyperspectral imaging. In practical agricultural scenarios, fluctuating sunlight, wind-induced leaf orientations, mixed stress, weed vegetation, or contamination of leaf surfaces can alter reflectance patterns and consequently lower the reliability of classification outcomes (Sankaran et al., 2011; Whetton et al., 2018; Abdulridha et al., 2020; Terentev et al., 2022). In addition, under real field conditions, stem rust infection usually develops primarily on stems rather than leaves (Singh et al., 2002; Kolmer, 2005), which may limit the visibility of characteristic spectral patterns and complicate disease detection. When adapting the proposed preprocessing and classification pipeline to UAV- or satellite-based observations, additional issues may arise, including spatial heterogeneity, instrument-specific noise, and mixed pixels that contain signals from both plant tissue and background elements (Adão et al., 2017; Qian, 2021; Zhang X. et al., 2024).

To determine the applicability of the developed pipeline to field conditions, additional testing is necessary, including evaluating the developed algorithms on the same disease in other crops, assessing applicability to other pathogens of the *Puccinia* family, and testing performance under mixed stress scenarios. Regarding the specific pathogenesis of stem rust - namely its predominant infection of stems - our approach captures spectral responses linked to early biochemical stress in the entire plant. It may therefore detect infection indirectly even before visible symptoms appear, an aspect that warrants further field verification. Results obtained by other teams assessing the severity of stem rust indicate that this does not present a serious obstacle to the application of hyperspectral sensing technology (Abdulridha et al., 2023).

A promising future direction for field application is the use of deep learning models. In recent years, deep learning methods, including one-dimensional and three-dimensional convolutional neural networks (1D-CNN and 3D-CNN), have become widely used in hyperspectral image classification tasks and have demonstrated high accuracy due to the extraction of complex spectral–spatial features. However, according to several modern benchmarks and studies (Nawar and Mouazen, 2017; Sudakov et al., 2019; Agata and Jaya, 2019; Jun, 2021; Ye et al., 2024), neural network models often fail to demonstrate significant improvement over boosting methods, while requiring orders of magnitude more computational resources and time for training and fine-tuning. Given that the goal of our study was to extensively test a wide range of preprocessing methods, computational efficiency was a key criterion for model selection. Tree-based methods allow efficient and rapid interpretation using gain-based split analysis or TreeSHAP (Lundberg et al., 2020), a fast variant of SHAP (Lundberg and Lee, 2017). Interpretation of neural networks, in turn, requires resource-intensive procedures based on multiple model evaluations, making the analysis significantly more labor-intensive (Molnar, 2020). Thus, based on requirements of computational efficiency and interpretability for a large-scale preprocessing evaluation, the following algorithms were selected as baselines. SVM: a classical method well established for plant disease hyperspectral classification (Rumpf et al., 2010; Baek et al., 2019; Guo et al., 2020); Logistic Regression: a linear model enabling assessment of feature space linear separability (Prabhakar et al., 2013; Mansournia et al., 2018); LGBM: a boosting model with high computational efficiency and generalization ability comparable to other boosting models (Chen and Guestrin, 2016; Prokhorenkova et al., 2018; George et al., 2024) and recent neural architectures (Golhani et al., 2018; Ye et al., 2024).

Nevertheless, we consider the use of deep methods an important direction for future research. Planned work includes experimental comparisons of various CNN architectures, as well as the exploration of models that combine the spectral–spatial strengths of neural networks with the interpretability and computational efficiency of classical approaches. These studies aim to expand the methodological foundation and improve the robustness of diagnostic models, especially as the problem setting approaches more complex, field-based conditions.

Overall, this study demonstrates that combining feature engineering (derivatives), robust preprocessing (particularly curve-wise normalization), and appropriate model selection enables reliable detection of wheat stem rust at an asymptomatic stage, even under challenging imaging conditions. Accuracy on day 4 post-inoculation reached 0.63–0.83, and on day 5 (still symptomless) 0.89–0.94, highlighting the potential of this approach for early disease management in precision agriculture. Future work will focus on (1) validating the approach across pathogens and crop species; (2) conducting full-scale field trials to assess generalization under natural variability; and (3) exploring advanced ML strategies, including ensemble learning and deep neural networks, to further enhance robustness and scalability.

## 5 Conclusions

This study presents the systematic comparison of pixel-, curve-, and channel-wise preprocessing strategies for early plant disease detection using hyperspectral imaging, demonstrated on wheat plants inoculated with *P. graminis* f. sp. *tritici*. Our results show that preprocessing choices substantially affect classification performance. The pipeline implementation enhanced the classification models accuracy raising F1-scores of machine learning methods from 0.67–0.75 (raw spectra) to 0.86–0.94 and enabled reliable detection of asymptomatic infections at 4 days after inoculation.

The proposed preprocessing pipeline can be used for clear, reproducible, and generalizable hyperspectral analysis of plant–pathogen interactions. Future work will focus on extending the approach to additional pathogens and further refining preprocessing strategies to improve early detection under diverse experimental conditions.

## Data Availability

The datasets presented in this study can be found in online repositories. The names of the repository/repositories and accession number(s) can be found below: https://drive.google.com/drive/folders/1vpKPlPw5uK5AnKctaE2oYCuOaRFX4-yN.

## References

[B1] Abdulridha J. Ampatzidis Y. Kakarla S. C. Robert P. (2020). Laboratory and UAV-based identification and classification of tomato yellow leaf curl, bacterial spot, and target spot diseases in tomato utilizing hyperspectral imaging and machine learning. Remote Sens. 12, 3843. doi: 10.3390/rs12213843

[B2] Abdulridha J. Min A. Rouse M. N. Kianian S. Isler V. Yang C. (2023). Evaluation of stem rust disease in wheat fields by drone hyperspectral imaging. Sensors 23, 4154. doi: 10.3390/s23084154, PMID: 37112495 PMC10141366

[B3] Adão T. Hruška J. Pádua L. Bessa J. Peres E. Morais R. . (2017). Hyperspectral imaging: A review on UAV-based sensors, data processing and applications for agriculture and forestry. Remote Sens. 9, p.1110. doi: 10.3390/rs9111110

[B4] Agata R. Jaya I. G. N. M. (2019). December. A comparison of extreme gradient boosting, SARIMA, exponential smoothing, and neural network models for forecasting rainfall data. J. Physics: Conf. Ser. 1397, 12073. doi: 10.1088/1742-6596/1397/1/012073

[B5] Akiba T. Sano S. Yanase T. Ohta T. Koyama M. (2019). “ Optuna: A next-generation hyperparameter optimization framework,” in Proceedings of the 25th ACM SIGKDD Conference on Knowledge Discovery and Data Mining (KDD 2019). (New York, NY, USA: ACM (Association for Computing Machinery)), 2623–2631. doi: 10.1145/3292500.3330701

[B6] Ashagre Z. A. (2022). Detection of wheat stem rust (Puccinia graminis f. sp. tritici) physiological races from major wheat producing regions of Ethiopia. Aquaculture Fisheries Stud. 4, 1–6. doi: 10.31038/AFS.2022433

[B7] Baek I. Kim M. S. Cho B. K. Mo C. Barnaby J. Y. McClung A. M. . (2019). Selection of optimal hyperspectral wavebands for detection of discolored, diseased rice seeds. Appl. Sci. 9, 1027. doi: 10.3390/app9051027

[B8] Baranova O. Solyanikova V. Kyrova E. Kon’kova E. Gaponov S. Sergeev V. . (2023). Evaluation of resistance to stem rust and identification of Sr genes in Russian spring and winter wheat cultivars in the Volga region. Agriculture 13, 635. doi: 10.3390/agriculture13030635

[B9] Bock C. H. Barbedo J. G. A. del Ponte E. M. Bohnenkamp D. Mahlein A. K. (2020). From visual estimates to fully automated sensor-based measurements of plant disease severity: Status and challenges for improving accuracy. Phytopathol. Res. 2, 9. doi: 10.1186/s42483-020-00057-8

[B10] Bohnenkamp D. Behmann J. Paulus S. Steiner U. Mahlein A. K. (2021). A hyperspectral library of foliar diseases of wheat. Phytopathology 111, 1583–1593. doi: 10.1094/PHYTO-12-20-0541-R, PMID: 33586995

[B11] Bohnenkamp D. Kuska M. T. Mahlein A. K. Behmann J. (2019). Hyperspectral signal decomposition and symptom detection of wheat rust disease at the leaf scale using pure fungal spore spectra as reference. Plant Pathol. 68, 1188–1195. doi: 10.1111/ppa.13020

[B12] Bolton M. D. Kolmer J. A. Garvin D. F. (2008). Wheat leaf rust caused by *Puccinia triticina*. Mol. Plant Pathol. 9, 563–575. doi: 10.1111/j.1364-3703.2008.00487.x, PMID: 19018988 PMC6640346

[B13] Bruning B. Berger B. Lewis M. Liu H. Garnett T. (2020). Approaches, applications, and future directions for hyperspectral vegetation studies: An emphasis on yield-limiting factors in wheat. Plant Phenome J. 3, e20007. doi: 10.1002/ppj2.20007

[B14] Chen T. Guestrin C. (2016). “ Xgboost: A scalable tree boosting system,” in Proceedings of the 22nd ACM SIGKDD Conference on Knowledge Discovery and Data Mining (KDD 2016). (New York, NY, USA: ACM (Association for Computing Machinery)), 785–794. doi: 10.1145/2939672.293978

[B15] Cortes C. Vapnik V. (1995). Support-vector networks. Mach. Learn. 20, 273–297. doi: 10.1007/BF00994018

[B16] Cozzolino D. Williams P. J. Hoffman L. C. (2023). An overview of pre-processing methods available for hyperspectral imaging applications. Microchemical J. 193, 109129. doi: 10.1016/j.microc.2023.109129

[B17] Defazio A. Bach F. Lacoste-Julien S. (2014). SAGA: A fast incremental gradient method with support for non-strongly convex composite objectives. Adv. Neural Inf. Process. Syst., 1646–1654. doi: 10.48550/arXiv.1407.0202

[B18] Deng J. Wang R. Yang L. Lv X. Yang Z. Zhang K. . (2023). Quantitative estimation of wheat stripe rust disease index using unmanned aerial vehicle hyperspectral imagery and innovative vegetation indices. IEEE Trans. Geosci. Remote Sens. 61, 1–11. doi: 10.1109/TGRS.2023.3292130

[B19] Fan R. E. Chang K. W. Hsieh C. J. Wang X. R. Lin C. J. (2008). LIBLINEAR: A library for large linear classification. J. Mach. Learn. Res. 9, 1871–1874.

[B20] FAO (Food and Agriculture Organization of the United Nations) (2025). The future of food and agriculture (Rome: FAO). Available online at: https://openknowledge.fao.org/ (Accessed November 18, 2025).

[B21] Friedman J. H. (2002). Stochastic gradient boosting. Comput. Stat Data Anal. 38, 367–378. doi: 10.1016/S0167-9473(01)00065-2

[B22] Fróna D. Szenderák J. Harangi-Rákos M. (2019). The challenge of feeding the world. Sustainability 11, 5816. doi: 10.3390/su11205816

[B23] Gao L. Smith R. T. (2015). Optical hyperspectral imaging in microscopy and spectroscopy: A review of data acquisition. J. Biophotonics 8, 441–456. doi: 10.1002/jbio.201400051, PMID: 25186815 PMC4348353

[B24] García-Vera Y. E. Polochè-Arango A. Mendivelso-Fajardo C. A. Gutiérrez-Bernal F. J. (2024). Hyperspectral image analysis and machine learning techniques for crop disease detection and identification: A review. Sustainability 16, 6064. doi: 10.3390/su16146064

[B25] George J. Yadav J. Nair A. M. (2024). “ September. Integrating lightGBM and XGBoost for robust plant disease classification: A homogenous stacking approach,” in Proceedings of the International Conference on Advances in Data-driven Computing and Intelligent Systems, Singapore. Springer Nature Singapore, 409–422. doi: 10.1007/978-981-96-5370-6_30

[B26] Golhani K. Balasundram S. K. Vadamalai G. Pradhan B. (2018). A review of neural networks in plant disease detection using hyperspectral data. Inf. Process. Agric. 5, 354–371. doi: 10.1016/j.inpa.2018.05.002

[B27] Grewal R. Singh Kasana S. Kasana G. (2023). Machine learning and deep learning techniques for spectral-spatial classification of hyperspectral images: A comprehensive survey. Electronics 12, 488. doi: 10.3390/electronics12030488

[B28] Grzybowski M. Wijewardane N. K. Atefi A. Ge Y. Schnable J. C. (2021). Hyperspectral reflectance-based phenotyping for quantitative genetics in crops: Progress and challenges. Plant Commun. 2, 100209. doi: 10.1016/j.xplc.2021.100209, PMID: 34327323 PMC8299078

[B29] Gu Q. Sheng L. Zhang T. Lu Y. Zhang Z. Zheng K. . (2019). Early detection of tomato spotted wilt virus infection in tobacco using the hyperspectral imaging technique and machine learning algorithms. Comput. Electron. Agric. 167, 105066. doi: 10.1016/j.compag.2019.105066

[B30] Guerri M. F. Distante C. Spagnolo P. Bougourzi F. Taleb-Ahmed A. (2024). Deep learning techniques for hyperspectral image analysis in agriculture: A review. ISPRS Open J. Photogrammetry Remote Sens. 12, 100062. doi: 10.1016/j.ophoto.2024.100062

[B31] Guo A. Huang W. Dong Y. Ye H. Ma H. Liu B. . (2021). Wheat yellow rust detection using UAV-based hyperspectral technology. Remote Sens. 13, 123. doi: 10.3390/rs13010123

[B32] Guo A. Huang W. Ye H. Dong Y. Ma H. Ren Y. . (2020). Identification of wheat yellow rust using spectral and texture features of hyperspectral images. Remote Sens. 12, 1419. doi: 10.3390/rs12091419

[B33] Hartigan J. A. Wong M. A. (1979). Algorithm AS 136: A k-means clustering algorithm. J. R. Stat. Society: Ser. C (Applied Statistics) 28, 100–108. doi: 10.2307/2346830

[B34] Hasan H. Shafri H. Z. M. Habshi M. (2019). A comparison between support vector machine (SVM) and convolutional neural network (CNN) models for hyperspectral image classification. IOP Conf. Series: Earth Environ. Sci. 357, 12035. doi: 10.1088/1755-1315/357/1/012035

[B35] Helin R. Indahl U. G. Tomic O. Liland K. H. (2022). On the possible benefits of deep learning for spectral preprocessing. J. Chemometrics 36, e3374. doi: 10.1002/cem.3374

[B36] Hosmer D. W. Lemeshow S. (2000). Applied logistic regression (Hoboken, NJ: John Wiley & Sons). doi: 10.1002/9781118548387

[B37] Huang L. Liu Y. Huang W. Dong Y. Ma H. Wu K. . (2022). Combining random forest and XGBoost methods in detecting early and mid-term winter wheat stripe rust using canopy-level hyperspectral measurements. Agriculture 12, 74. doi: 10.3390/agriculture12010074

[B38] Jacquemoud S. Ustin S. (2019). Leaf optical properties (Cambridge, United Kingdom: Cambridge University Press). doi: 10.1017/9781108686457

[B39] Jin Y. Singh R. P. Ward R. W. Wanyera R. Kinyua M. Njau P. . (2007). Characterization of seedling infection types and adult plant infection responses of monogenic Sr gene lines to race TTKS of Puccinia graminis f. sp. tritici. Plant Dis. 91, 1096–1099. doi: 10.1094/PDIS-91-9-1096, PMID: 30780647

[B40] Jun M. J. (2021). A comparison of a gradient boosting decision tree, random forests, and artificial neural networks to model urban land use changes: The case of the Seoul metropolitan area. Int. J. Geographical Inf. Sci. 35, 2149–2167. doi: 10.1080/13658816.2021.1887490

[B41] Ke G. Meng Q. Finley T. Wang T. Chen W. Ma W. . (2017). “ LightGBM: A highly efficient gradient boosting decision tree,” in Proceedings of the 31st Conference on Neural Information Processing Systems (NeurIPS 2017). (Red Hook, NY, USA: Curran Associates, Inc.), 3146–3154. doi: 10.48550/arXiv.1712.01012

[B42] Khaled A. A. Reda O. I. Yaser H. M. Esmail S. M. El Sabagh A. (2018). Anatomical, biochemical and physiological changes in some Egyptian wheat cultivars inoculated with *Puccinia graminis* f. sp. *tritici*. Fresenius Environ. Bull. 27, 296–305.

[B43] Khan A. Vibhute A. D. Mali S. Patil C. H. (2022). A systematic review on hyperspectral imaging technology with a machine and deep learning methodology for agricultural applications. Ecol. Inf. 69, 101678. doi: 10.1016/j.ecoinf.2022.101678

[B44] Kolmer J. A. (2005). Tracking wheat rust on a continental scale. Curr. Opin. Plant Biol. 8, 441–449. doi: 10.1016/j.pbi.2005.05.001, PMID: 15922652

[B45] Kolmer J. (2013). Leaf rust of wheat: pathogen biology, variation and host resistance. Forests 4, 70–84. doi: 10.3390/f4010070

[B46] Kolmer J. A. (2019). Virulence of Puccinia triticina, the wheat leaf rust fungus, in the United States in 2017. Plant Dis. 103, 2113–2120. doi: 10.1094/PDIS-10-18-1829-RE 31161933

[B47] Kolmer J. A. Jin Y. Long D. L. (2007). Wheat leaf and stem rust in the United States. Aust. J. Agric. Res. 58, 631–638. doi: 10.1071/AR07159

[B48] Leonard K. J. Szabo L. J. (2005). Pathogen profile: Stem rust of small grains and grasses caused by *Puccinia graminis*. Mol. Plant Pathol. 6, 99–111. doi: 10.1111/j.1364-3703.2005.00273.x, PMID: 20565642

[B49] Lewis C. M. Persoons A. Bebber D. P. Kigathi R. N. Maintz J. Findlay K. . (2018). Potential for re-emergence of wheat stem rust in the United Kingdom. Commun. Biol. 1, 13. doi: 10.1038/s42003-018-0015-7, PMID: 30271900 PMC6053080

[B50] Li Y.-h. Tan X. Zhang W. Jiao Q.-b. Xu Y.-x. Li H. . (2021). Research and application of several key techniques in hyperspectral image preprocessing. Front. Plant Sci. 12. doi: 10.3389/fpls.2021.627865, PMID: 33679841 PMC7935556

[B51] Liu Z. Li H. Li Y. Xu W. Sun Y. (2023). Hyperspectral imaging for early cancer detection: Recent advances and future trends. Cancers 15, 5041. doi: 10.3390/cancers15205041, PMID: 37894408 PMC10605500

[B52] Lundberg S. M. Erion G. Chen H. DeGrave A. Prutkin J. M. Nair B. . (2020). From local explanations to global understanding with explainable AI for trees. Nat. Mach. Intell. 2, 56–67. doi: 10.1038/s42256-019-0138-9, PMID: 32607472 PMC7326367

[B53] Lundberg S. M. Lee S. I. (2017). A unified approach to interpreting model predictions. Adv. Neural Inf. Process. Syst. 30. doi: 10.48550/arXiv.1705.07874

[B54] Mahlein A. K. (2016). Plant disease detection by imaging sensors–parallels and specific demands for precision agriculture and plant phenotyping. Plant Dis. 100, 241–251. doi: 10.1094/PDIS-03-15-0340-FE, PMID: 30694129

[B55] Mansournia M. A. Geroldinger A. Greenland S. Heinze G. (2018). Separation in logistic regression: causes, consequences, and control. Am. J. Epidemiol. 187, pp.864–pp.870. doi: 10.1093/aje/kwx299, PMID: 29020135

[B56] McDonald J. H. (2014). Handbook of biological statistics (Maryland: Sparky House Publishing, Baltimore). Available online at: https://www.biostathandbook.com/HandbookBioStatThird.pdf (Accessed November 18, 2025).

[B57] Meher S. K. Panda G. (2021). Deep learning in astronomy: A tutorial perspective. Eur. Phys. J. Special Topics 230, 2285–2317. doi: 10.1140/epjs/s11734-021-00136-9

[B58] Molnar C. (2020). Interpretable machine learning. Available online at: https://christophm.github.io/interpretable-ml-book/ (Accessed November 18, 2025).

[B59] Nawar S. Mouazen A. M. (2017). Comparison between random forests, artificial neural networks and gradient boosted machines methods of on-line Vis-NIR spectroscopy measurements of soil total nitrogen and total carbon. Sensors 17, 2428. doi: 10.3390/s17102428, PMID: 29064411 PMC5677209

[B60] Olivera Firpo P. D. Newcomb M. Flath K. Sommerfeldt-Impe N. Szabo L. J. Carter M. . (2017). Characterization of Puccinia graminis f. sp. tritici isolates derived from an unusual wheat stem rust outbreak in Germany in 2013. Plant Pathol. 66, 1258–1266. doi: 10.1111/ppa.12683

[B61] Paoletti M. Haut J. Plaza J. Plaza A. (2019). Deep learning classifiers for hyperspectral imaging: A review. ISPRS J. Photogrammetry Remote Sens. 158, 279–317. doi: 10.1016/j.isprsjprs.2019.09.006

[B62] Patpour M. Hovmøller M. S. Rodriguez-Algaba J. Randazzo B. Villegas D. Shamanin V. P. . (2022). Wheat stem rust back in Europe: Diversity, prevalence and impact on host resistance. Front. Plant Sci. 13. doi: 10.3389/fpls.2022.882440, PMID: 35720526 PMC9202592

[B63] Peterson R. F. Campbell A. B. Hannah A. E. (1948). A diagrammatic scale for estimating rust intensity on leaves and stems of cereals. Can. J. Res. 26, 496–500. doi: 10.1139/cjr48c-033

[B64] Prabhakar M. Prasad Y. G. Desai S. Thirupathi M. Gopika K. Rao G. R. . (2013). Hyperspectral remote sensing of yellow mosaic severity and associated pigment losses in Vigna mungo using multinomial logistic regression models. Crop Prot. 45, 132–140. doi: 10.1016/j.cropro.2012.12.003

[B65] Pretorius Z. A. Singh R. P. Wagoire W. W. Payne T. S. (2000). Detection of virulence to wheat stem rust resistance gene Sr31 in Puccinia graminis f. sp. tritici in Uganda. Plant Dis. 84, 203. doi: 10.1094/PDIS.2000.84.2.203B, PMID: 30841334

[B66] Prokhorenkova L. Gusev G. Vorobev A. Dorogush A. V. Gulin A. (2018). CatBoost: unbiased boosting with categorical features. Adv. Neural Inf. Process. Syst. 31. doi: 10.48550/arXiv.1706.09516

[B67] Pu R. (2017). Hyperspectral Remote Sensing: Fundamentals and Practices (Boca Raton, FL: CRC Press). doi: 10.1201/9781315152223

[B68] Qi C. Sandroni M. Westergaard J. C. Sundmark E. Bagge M. Alexandersson E. . (2021). In-field early disease recognition of potato late blight based on deep learning and proximal hyperspectral imaging. arXiv:2111.12155.

[B69] Qi C. Sandroni M. Westergaard J. C. Sundmark E. H. R. Bagge M. Alexandersson E. . (2023). In-field classification of the asymptomatic biotrophic phase of potato late blight based on deep learning and proximal hyperspectral imaging. Comput. Electron. Agric. 205, 107585. doi: 10.1016/j.compag.2023.107585

[B70] Qian S. E. (2021). Hyperspectral satellites, evolution, and development history. IEEE J. Selected Topics Appl. Earth Observations Remote Sens. 14, 7032–7056. doi: 10.1109/JSTARS.2021.3090256

[B71] Radočaj D. Šiljeg A. Marinović R. Jurišić M. (2023). State of major vegetation indices in precision agriculture studies indexed in Web of Science: A review. Agriculture 13, 707. doi: 10.3390/agriculture13030707

[B72] Ren K. Guo A. Qian B. Ruan C. Huang W. Dong Y. . (2025). Inversion of plant functional traits from hyperspectral imagery enhances the distinction of wheat stripe rust severity. Artif. Intell. Agriculture 16, 206–223. doi: 10.1016/j.aiia.2025.10.006

[B73] Rinnan >Å. Van den Berg F. Engelsen S. B. (2009). Review of the most common pre-processing techniques for near-infrared spectra. TrAC Trends Analytical Chem. 28, 1201–1222. doi: 10.1016/j.trac.2009.07.007

[B74] Rossel R. A. V. Lobsey C. Sharman C. McLachlan G. Padarian J. (2017). Novel proximal soil sensing instrument for rapid in situ measurement of soil pH. Sensors 17, 2489. doi: 10.3390/s17112489, PMID: 29084158 PMC5712851

[B75] Rumpf T. Mahlein A. K. Steiner U. Oerke E. C. Dehne H. W. Plümer L. (2010). Early detection and classification of plant diseases with support vector machines based on hyperspectral reflectance. Comput. Electron. Agric. 74, 91–99. doi: 10.1016/j.compag.2010.06.009

[B76] Sankaran S. Mishra A. Maja J. M. Ehsani R. (2011). Visible–near infrared spectroscopy for detection of Huanglongbing (HLB) using a VIS-NIR spectroscopy technique. Comput. Electron. Agric. 77, 127–134. doi: 10.1016/j.compag.2011.03.002

[B77] Savitzky A. Golay M. J. E. (1964). Smoothing and differentiation of data by simplified least squares procedures. Analytical Chem. 36, 1627–1639. doi: 10.1021/ac60214a047

[B78] Schumann G. L. Leonard K. J. (2000). Stem rust of wheat. Plant Health Instructor 58, 1–12. doi: 10.1094/PHI-I-2000-0721-01

[B79] Shahi T. B. Xu C.-Y. Neupane A. Guo W. (2023). Recent advances in crop disease detection using UAV and deep learning techniques. Remote Sens. 15, 2450. doi: 10.3390/rs15102450

[B80] Singh R. P. Huerta-Espino J. Roelfs A. P. Curtis B. C. (2002). The wheat rusts. Growth 2, 35.

[B81] St L. Wold S. (1989). Analysis of variance (ANOVA). Chemometrics intelligent Lab. Syst. 6, 259–272. doi: 10.1016/0169-7439(89)80095-4

[B82] Stakman E. C. Stewart D. M. Loegering W. Q. (1962). Identification of physiologic races of Puccinia graminis var. tritici (Washington, DC, USA: Agricultural Research Service E617. United States Department of Agriculture).

[B83] Stuart M. B. McGonigle A. J. S. Willmott J. R. (2019). Hyperspectral imaging in environmental monitoring: A review of recent developments and technological advances in compact field deployable systems. Sensors 19, 3071. doi: 10.3390/s19143071, PMID: 31336796 PMC6678368

[B84] Sudakov O. Burnaev E. Koroteev D. (2019). Driving digital rock towards machine learning: Predicting permeability with gradient boosting and deep neural networks. Comput. geosciences 127, 91–98. doi: 10.1016/j.cageo.2019.02.002

[B85] Terentev A. Badenko V. Shaydayuk E. Emelyanov D. Eremenko D. Klabukov D. . (2023). Hyperspectral remote sensing for early detection of wheat leaf rust caused by Puccinia triticina. Agriculture 13, 1186. doi: 10.3390/agriculture13061186

[B86] Terentev A. Dolzhenko V. (2023). Can metabolomic approaches become a tool for improving early plant disease detection and diagnosis with modern remote sensing methods? A review. Sensors 23, 5366. doi: 10.3390/s23125366, PMID: 37420533 PMC10302926

[B87] Terentev A. Dolzhenko V. Fedotov A. Eremenko D. (2022). Current state of hyperspectral remote sensing for early plant disease detection: A review. Sensors 22, 757. doi: 10.3390/s22030757, PMID: 35161504 PMC8839015

[B88] Terentev A. Kuznetsova D. Fedotov A. Baranova O. Eremenko D. (2025). Cross-crop transferability of machine learning models for early stem rust detection in wheat and barley using hyperspectral imaging. Plants 14, 3265. doi: 10.3390/plants14213265, PMID: 41225815 PMC12608451

[B89] Thapa S. Gill H. S. Halder J. Rana A. Ali S. Maimaitijiang M. . (2024). Integrating genomics, phenomics, and deep learning improves the predictive ability for Fusarium head blight–related traits in winter wheat. Plant Genome 17, e20470. doi: 10.1002/tpg2.20470, PMID: 38853339 PMC12807346

[B90] Ube N. Harada D. Katsuyama Y. Osaki-Oka K. Tonooka T. Ueno K. . (2019). Identification of phenylamide phytoalexins and characterization of inducible phenylamide metabolism in wheat. Phytochemistry 167, 112098. doi: 10.1016/j.phytochem.2019.112098, PMID: 31450090

[B91] Virtanen P. Gommers R. Oliphant T. E. Haberland M. Reddy T. Cournapeau D. . (2020). SciPy 1.0: Fundamental algorithms for scientific computing in Python. Nat. Methods 17, 261–272. doi: 10.1038/s41592-019-0686-2, PMID: 32015543 PMC7056644

[B92] Wan L. Li H. Li C. Wang A. Yang Y. Wang P. (2022). Hyperspectral sensing of plant diseases: Principle and methods. Agronomy 12, 1451. doi: 10.3390/agronomy12061451

[B93] Wang D. Vinson R. Holmes M. Seibel G. Bechar A. Nof S. . (2018). “ Early tomato spotted wilt virus detection using hyperspectral imaging technique and outlier removal auxiliary classifier generative adversarial nets (OR-AC-GAN),” in Proceedings of the 2018 ASABE Annual International Meeting. St. Joseph, Michigan, USA: American Society of Agricultural and Biological Engineers, 1. doi: 10.13031/aim.201800660, PMID:

[B94] Whetton R. L. Waine T. W. Mouazen A. M. (2018). Hyperspectral measurements of yellow rust and Fusarium head blight in cereal crops: Part 2—On-line field measurement. Biosyst. Eng. 167, 144–158. doi: 10.1016/j.biosystemseng.2017.07.012

[B95] Willocquet L. Rossi V. Savary S. (2022). Simulation modelling of yield losses caused by wheat stem rust. Plant Pathol. 71, 544–555. doi: 10.1111/ppa.13488

[B96] Witteveen M. Sterenborg H. J. van Leeuwen T. G. Aalders M. C. Ruers T. J. Post A. L. (2022). Comparison of preprocessing techniques to reduce nontissue-related variations in hyperspectral reflectance imaging. J. Biomed. Optics 27, 106003. doi: 10.1117/1.JBO.27.10.106003, PMID: 36207772 PMC9541333

[B97] Xu X. Chen S. Xu Z. Yu Y. Zhang S. Dai R. (2020). Exploring appropriate preprocessing techniques for hyperspectral soil organic matter content estimation in black soil area. Remote Sens. 12, 3765. doi: 10.3390/rs12223765

[B98] Ye H. J. Liu S. Y. Cai H. R. Zhou Q. L. Zhan D. C. (2024). A closer look at deep learning methods on tabular datasets. arXiv preprint arXiv:2407.00956. doi: 10.48550/arXiv.2407.00956

[B99] Yoon J. (2022). Hyperspectral imaging for clinical applications. BioChip J. 16, 1–12. doi: 10.1007/s13206-021-00046-z

[B100] Zhang Z. Huang L. Wang Q. Jiang L. Qi Y. Wang S. . (2024). UAV hyperspectral remote sensing image classification: A systematic review. IEEE J. Selected Topics Appl. Earth Observations Remote Sens. 18, 3099–3123. doi: 10.1109/JSTARS.2024.3522318

[B101] Zhang Y. Li X. Wang M. Xu T. Huang K. Sun Y. . (2024). Early detection and lesion visualization of pear leaf anthracnose based on multi-source feature fusion of hyperspectral imaging. Front. Plant Sci. 15, 1461855. doi: 10.3389/fpls.2024.1461855, PMID: 39439506 PMC11493603

[B102] Zhang X. Vinatzer B. A. Li S. (2024). Hyperspectral imaging analysis for early detection of tomato bacterial leaf spot disease. Sci. Rep. 14, 27666. doi: 10.1038/s41598-024-78650-6, PMID: 39532930 PMC11557939

[B103] Zhang N. Yang G. Pan Y. Yang X. Chen L. Zhao C. (2020). A review of advanced technologies and development for hyperspectral-based plant disease detection in the past three decades. Remote Sens. 12, 3188. doi: 10.3390/rs12193188

[B104] Zhu H. Cen H. Zhang C. He Y. (2016). “ Early detection and classification of tobacco leaves inoculated with tobacco mosaic virus based on hyperspectral imaging technique,” in Proceedings of the 2016 ASABE Annual International Meeting. St. Joseph, Michigan, USA: American Society of Agricultural and Biological Engineers, 1. doi: 10.13031/aim.20162460422

